# Targeted Deficiency of the Transcriptional Activator Hnf1α Alters Subnuclear Positioning of Its Genomic Targets

**DOI:** 10.1371/journal.pgen.1000079

**Published:** 2008-05-23

**Authors:** Reini F. Luco, Miguel A. Maestro, Nicolas Sadoni, Daniele Zink, Jorge Ferrer

**Affiliations:** 1 Genomic Programming of Beta-cells Laboratory, Institut d'Investigacions August Pi i Sunyer, Barcelona, Spain; 2 CIBER de Diabetes y Enfermedades Metabólicas Asociadas (CIBERDEM), Barcelona, Spain; 3 Department of Biology II, Ludwig Maximilians University Munich, Planegg-Martinsried, Germany; 4 Visitron Systems GmbH, Puchheim, Germany; 5 Institute of Bioengineering and Nanotechnology, The Nanos, Singapore; 6 Endocrinology, Hospital Clinic de Barcelona, Barcelona, Spain; MRC Human Genetics Unit, United Kingdom

## Abstract

DNA binding transcriptional activators play a central role in gene-selective regulation. In part, this is mediated by targeting local covalent modifications of histone tails. Transcriptional regulation has also been associated with the positioning of genes within the nucleus. We have now examined the role of a transcriptional activator in regulating the positioning of target genes. This was carried out with primary β-cells and hepatocytes freshly isolated from mice lacking Hnf1α, an activator encoded by the most frequently mutated gene in human monogenic diabetes (MODY3). We show that in *Hnf1a^−/−^* cells inactive endogenous Hnf1α-target genes exhibit increased trimethylated histone H3-Lys27 and reduced methylated H3-Lys4. Inactive Hnf1α-targets in *Hnf1a^−/−^* cells are also preferentially located in peripheral subnuclear domains enriched in trimethylated H3-Lys27, whereas active targets in wild-type cells are positioned in more central domains enriched in methylated H3-Lys4 and RNA polymerase II. We demonstrate that this differential positioning involves the decondensation of target chromatin, and show that it is spatially restricted rather than a reflection of non-specific changes in the nuclear organization of *Hnf1a*-deficient cells. This study, therefore, provides genetic evidence that a single transcriptional activator can influence the subnuclear location of its endogenous genomic targets in primary cells, and links activator-dependent changes in local chromatin structure to the spatial organization of the genome. We have also revealed a defect in subnuclear gene positioning in a model of a human transcription factor disease.

## Author Summary

All cells in an organism share a common genome, yet distinct subsets of genes are transcribed in different cells. Selectivity of gene transcription is largely determined by transcription factors that bind to target genes and promote local changes in chromatin. Such changes are thought to be instrumental for transcription. Emerging evidence indicates that the position of genes in the 3-dimensional structure of the nucleus may also be important in transcriptional regulation. However, the role of transcription factors in gene positioning, and its possible relationship with chromatin modifications, is poorly understood. To examine this, we employed a genetic approach. We used mice lacking Hnf1α, a transcription factor gene that is mutated in an inherited form of diabetes. We studied genes that are directly bound by Hnf1α, as well as various control genomic regions, and determined their position in nuclear space in liver and insulin-producing β-cells. The results showed that the absence of Hnf1α causes local changes in the chromatin of target genes. At the same time, it modifies the position of target genes in nuclear space. The findings of this study lead us to propose a model whereby transcription factor dependent local chromatin modifications are linked to subnuclear gene positioning. They also revealed abnormal subnuclear positioning in a model of a human transcription factor disease.

## Introduction

The recognition of nucleotide sequences in the vicinity of genes by DNA binding factors is central to the regulation of gene-specific transcription [1]. The mechanism by which DNA binding transactivators lead to gene activation is in part dependent on their ability to promote the remodeling of chromatin structure and the covalent modification of nucleosomal histone tails [1]–[3].

Numerous studies have linked different covalent histone modifications with the transcriptional state of gene loci [4]. Amongst these, the methylation of H3-Lys4 at gene promoters has been linked to gene activity [5],[6], whereas transcriptional silencing correlates with increased methylation of H3-Lys9 or H3-Lys27 [7]–[10]. For example, trimethylated histone H3 Lysine 9 (H3-Lys9me3) is enriched at pericentromeric repeats forming constitutive heterochromatin [9],[11], while trimethylated H3-Lys27 (H3-Lys27me3) has been linked to other forms of inactive chromatin, including chromosome X facultative heterochromatin, imprinted loci, and Polycomb-mediated silencing of homeobox gene clusters [7],[8],[10].

It has recently become apparent that the positioning of gene loci within the three dimensional structure of the nucleus may provide a further level of regulation (reviewed in [2],[12],[13]. Gene activation has been linked to selective looping of loci away from chromosome territories [14],[15], and appears to be associated with an increased likelihood that a locus intermingles with heterologous chromosome territories [16]. Transcribing genes have also been shown to colocalize with nuclear domains that are visibly enriched in RNA polymerase II [17],[18]. Other observations revealed that active loci localize in the nuclear interior, whereas inactive genes have been found to be preferentially positioned at the nuclear periphery [19],[20]. Moreover, repositioning to centromeric regions has been shown for several hematopoietic genes during differentiation-related silencing, and correlates with mutation-induced silencing of the brown locus in Drosophila [21]–[24].

The precise relationships between gene positioning and transcriptional regulation, however, are not understood. Some studies suggest that gene compartmentalization may play a decisive regulatory role. An example is the demonstration that artificial recruitment of genes to the nuclear lamina results in transcriptional repression in certain, though not all, experimental settings [25],[26]. On the other hand, several studies show that gene-rich regions tend to locate outside of their respective chromosome territories or occupy more central nuclear positions [27]–[30]. Therefore, the extent to which a gene's subnuclear position in a given cell type depends on its gene-specific transcriptional activity or on the regional organization of the chromosome territory is unclear.

Furthermore, little is known about the mechanisms that govern gene positioning, or the possible role of transcriptional activators in this process. Recent studies have shown that fusion proteins containing activation domains can cause dynamic subnuclear relocation of artificial multicopy genomic targets [31],[32]. However, so far, no study has addressed the role of endogenous activators in the positioning of endogenous genes. Furthermore, the possible interplay of gene positioning with other activator-dependent effects, such as site-specific chromatin modifications, is poorly understood.

In the current study, we explored the relationships between transcriptional activator function, chromatin structure, and subnuclear gene positioning. This was addressed using mice with targeted ablation of the *Hnf1a* gene (also known as *Tcf1*). Hnf1α (Hepatocyte nuclear factor 1α) is a homeodomain protein encoded by the gene implicated in MODY3 (Maturity-onset diabetes of the young 3), the most common form of human monogenic diabetes [33]. Studies performed with knock-out mice have shown that Hnf1α is dispensable for organogenesis, but is essential for the activity of several direct target genes involved in differentiated functions of liver, kidney, and pancreatic β-cells [34]–[38]. Using immuno-FISH, we studied the subnuclear position of endogenous direct targets of Hnf1α in freshly isolated primary hepatocytes and islet-cells from *Hnf1a-*null mutant vs. control mice. This model enabled us to ascribe observed changes to the presence or absence of an activator, in contrast to previous studies comparing the position of a locus among cell-types or developmental stages which potentially differ markedly in their chromosomal configurations [39]. At the same time, this model overcomes the limitations of using transformed cell lines and artificial overexpression systems.

The results provide genetic evidence that a transcriptional activator influences the subnuclear position of its endogenous genomic targets in primary cells. In addition, we present data to support that activator-dependent changes in local histone modifications and chromatin condensation may play a role in regulating the spatial organization of the cell nucleus. Collectively, the results provide novel insights into the *in vivo* functions of a transcriptional activator and increase our understanding of the cellular defects underlying a human transcriptional disease.

## Results

### Hnf1α Alters Histone Methylation Patterns and Chromatin Compaction of Its Target Genes

**Figure 1:**
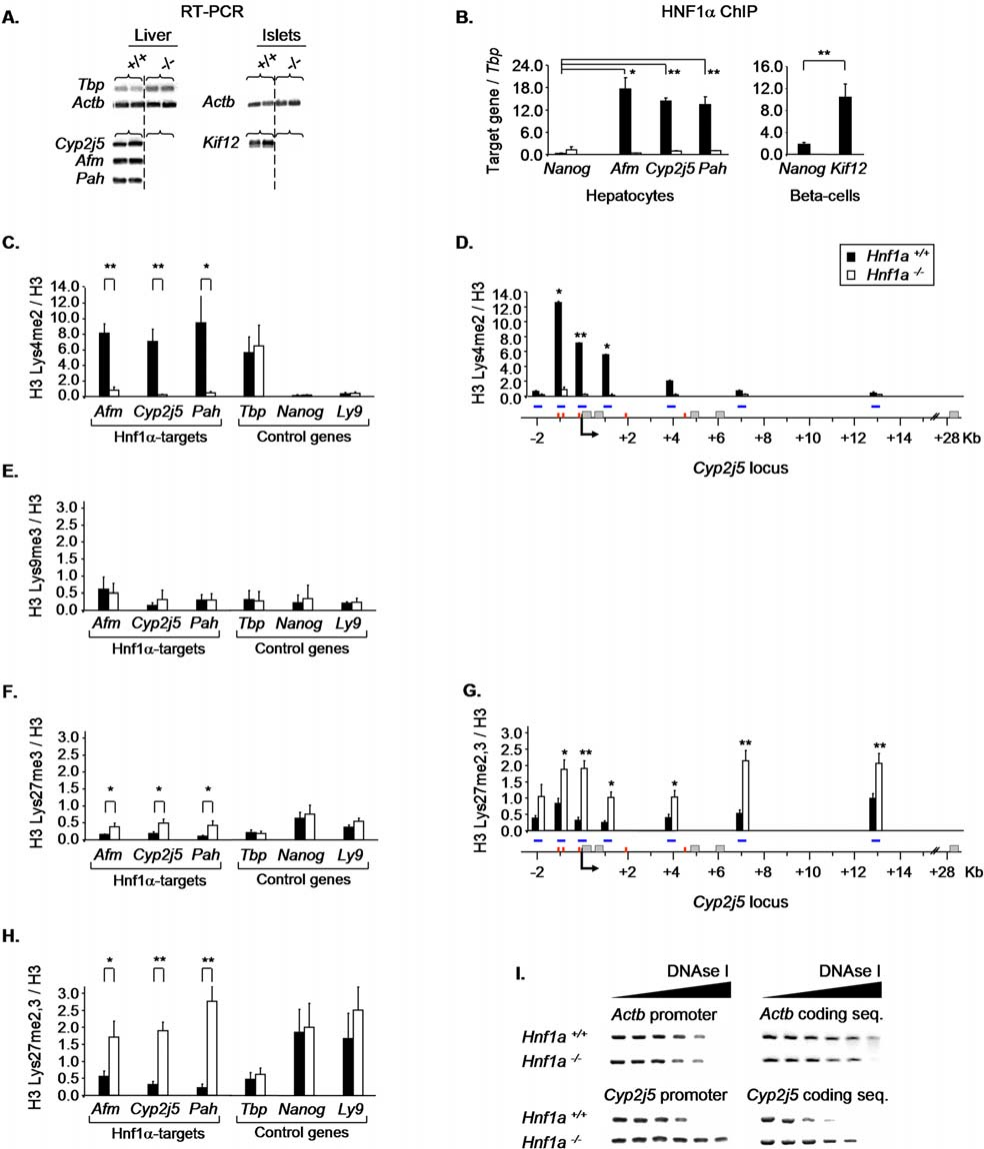
Inactive *Hnf1α* targets in *Hnf1a^−/−^* cells exhibit local enrichment of H3-Lys27me3, decreased H3-Lys4me2, and reduced DNAse I sensitivity. (A) RNA expression of tissue-specific *Hnf1α* targets (*Afm, Cyp2j5, Pah, Kif12*) and control genes (*Tbp, Actb*) in *Hnf1a^+/+^* and *Hnf1a^−/−^* liver and islets. (B) ChIP analysis of Hnf1α occupancy in the promoter region of tissue-specific targets and a control gene (*Nanog*) in *Hnf1a^+/+^* and *Hnf1a^−/−^* hepatocytes (*Afm, Cyp2j5 and Pah*, black and white bars respectively) and MIN6 beta-cells (*Kif12*, black bars). Results are normalized by *Tbp* enrichment. *p<0.05 and **p<0.01 relative to *Nanog.* (C–H) ChIP analysis of histone modifications in Hnf1α-targets and control genes in *Hnf1a^+/+^* and *Hnf1a^−/−^* hepatocytes. For all genes the 5′ flanking regions are analyzed, except in D,G, where the entire *Cyp2j5* locus is analyzed. Blue horizontal lines indicate amplicon positions, grey boxes are exons, red lines depict computationally predicted high-affinity HNF1 binding sites, and an arrow indicates the transcription start site. Graphs depict mean±SEM of the ratio of the percent input immunoprecipitated with anti-methyl specific H3 relative to anti-H3 antibodies in 3 independent experiments. Black bars represent *Hnf1a^+/+^* and white bars *Hnf1a^−/−^* hepatocytes. *p<0.05 and **p<0.01 relative to *Hnf1a^+/+^* hepatocytes. (I) General DNAse sensitivity of *Cyp2j5*. Representative PCR analysis of *Cyp2j5* and *Actb* 5′ flanking and coding sequence regions after digestion of *Hnf1a^+/+^* and *Hnf1a^−/−^* nuclei with increasing amounts of DNAse I. Results show reduced DNAse I sensitivity in *Hnf1a^−/−^* nuclei in the *Cyp2j5* region, but not in the *Actb* control locus.

Earlier studies showed that Hnf1α-dependent transcription is dependent on the recruitment of histone acetyltransferases and the local acetylation of nucleosomal histones [40],[41]. We have now examined the methylation state of histone H3 in target genes. For this analysis we selected the most profoundly downregulated genes identified in expression profiling experiments of *Hnf1a^−/−^* hepatocytes (*Afm*, *Cyp2j5* and *Pah*) and islets (*Kif12*), all of which are specifically downregulated in their respective Hnf1α-deficient cell-types (Figure 1A). The four genes contain evolutionary conserved high-affinity Hnf1 binding sites in their promoter regions, and were experimentally shown to be directly bound by Hnf1α (Figure 1B and not shown).

As shown in Figure 1C–D, dimethylated H3-Lys4 (H3-Lys4me2) was decreased in the 5′ region of such genes in hepatocytes from *Hnf1a*-deficient mice, while no changes were observed in control genes.

H3-Lys9me3, an established repressive mark associated with constitutive heterochromatin [9], was not increased in the 5′ region of these genes in *Hnf1a^−/−^* hepatocytes (Figure 1E), but was readily detected in minor satellite positive control sequences (data not shown).

In contrast, methylated H3-Lys27 was increased in Hnf1α-dependent targets in *Hnf1a^−/−^* hepatocytes to a similar extent as in two constitutively silenced genes known to be enriched in this repressive mark (*Nanog* and *Hoxa9)*, whereas no changes were observed in non Hnf1α-dependent control genes (Figure 1F–H and not shown). Increased methylated H3-Lys27 was primarily the trimethylated form, as it was detected with selective antisera for H3-Lys27me2,3 and H3-Lys27me3, but not H3-Lys27me2 (Figure 1F–H and not shown). Interestingly, increased H3-Lys27me2,3 was spread throughout the *Cyp2j5* locus, rather than being circumscribed to discrete segments (Figure 1H). Dimethylated H3-Lys9, another histone mark previously associated with facultative heterochromatin, was also increased by 3.5 to 5-fold in inactive Hnf1α-targets in *Hnf1a^−/−^* cells (data not shown).

We also examined the consequences of Hnf1α-deficiency on target chromatin condensation. General DNAse I sensitivity studies revealed reduced degradation of *Cyp2j5* chromatin in *Hnf1a^−/−^* vs. *Hnf1a^+/+^* hepatocytes, whereas no differences were observed between genotypes for the control gene *Actb* (Figure 1I). Thus, in direct Hnf1α target genes that are inactive due to Hnf1α-deficiency, there is a switch from an active chromatin conformation enriched in methylated H3-Lys4, to a more compacted state enriched in trimethylated H3-Lys27.

### Different Isoforms of Methylated Histone H3 Are Distributed Non-Randomly in Nuclear Space

**Figure 2:**
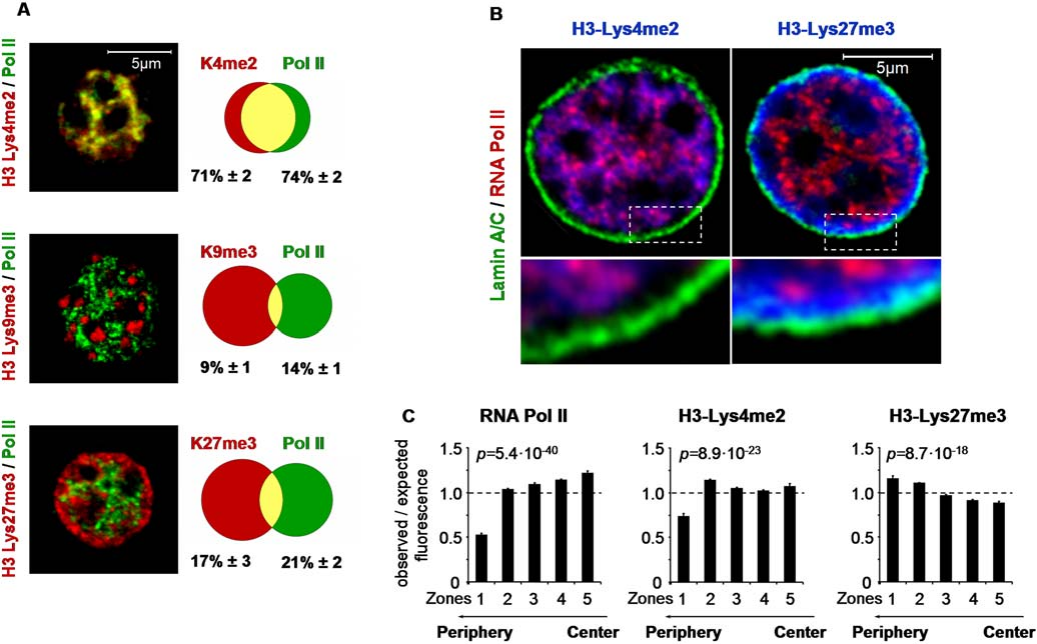
Methylated histone H3 marks exhibit a non-random subnuclear distribution. (A) Dual immunofluorescence confocal analysis of domains enriched in H3-Lys4me2, H3-Lys9me3, and H3-Lys27me3 (red) compared with RNA polymerase II (green) in interphase wild-type hepatocyte nuclei. Adjacent Venn diagrams display percentages of colocalization (mean±SEM from 20 nuclei) of signals exceeding the 75^th^ percentile of nuclear signal intensity in wild-type nuclei. (B) Triple immunofluorescence of RNA polymerase II (red), lamin A/C (green) and either H3-Lys4me2 or H3-Lys27me3 (blue) in hepatocytes. Insets below show peripheral nuclear segments at higher magnification. (C) Erosion analysis of the nuclear distribution of RNA polymerase II, H3-Lys4me2, or H3-Lys27me3. Nuclei were subdivided into 5 concentric zones, and total nuclear fluorescence intensities were determined for each epitope using non-thresholded images. The graphs indicate the percentages of total nuclear fluorescence intensities observed in each zone (mean±SEM). The values were normalized to the relative nuclear areas occupied by the different zones, so that a value of 1 was obtained if the percentage is as expected in case of unbiased distribution. At least 20 nuclei were analyzed in each case. Significance values for the comparison between the 5 zones for each of the 3 epitopes were obtained by ANOVA.

To explore possible relationships between Hnf1α-dependent gene activity, site-specific histone modifications, and nuclear organization, we first assessed subnuclear distributions of histone modifications in primary hepatocytes and pancreatic islet-cells. Both of these cell types are largely quiescent under normal conditions. The results showed that H3-Lys4me2-rich subnuclear regions displayed a high degree of colocalization with regions that are enriched in RNA polymerase II phosphorylated on serine 5 of the C terminal repeat, the predominant polymerase form in the transcriptional initiation complex [42] (hereafter referred to as RNA polymerase II) (Figure 2A). In sharp contrast, gene-silencing marks H3-Lys9me3 and Lys27me3 were more abundant in regions that were not enriched in RNA polymerase II (Figure 2A). These subnuclear distributions were independent of the fixative and processing methods used, and were observed with different H3-Lys27me3 antibodies (Figure S1A,B). Furthermore, the H3-Lys27me3 immunostaining pattern was distinct from that of Histone H3 and other modifications including H3-Lys4me2, H3-Lys27me1, H3-Lys27me2, H3-Lys9me3, as well as the DNA stain TO-PRO-3, indicating that it does not merely reflect chromatin density (Figure S1C–F, Figure S2, and not shown).

We next examined the radial distribution of histone modifications. H3-Lys27me3 was markedly enriched whereas H3-Lys4me2 displayed relative depletion in the immediate vicinity of the inner nuclear membrane, as shown by co-immunostaining of Lamin A/C (Figure 2B). Erosion analyses using non-thresholded images furthermore revealed markedly different radial enrichment patterns for RNA polymerase II, H3-Lys4me2, and H3-Lys27me3 (Figure 2C). Thus, RNA polymerase II and H3-Lys4me2 were significantly depleted in peripheral nuclear zones compared to more interior nuclear regions (Figure 2C, ANOVA p values 5.4×10^−40^ and 8.9×10^−23^). In contrast, H3-Lys27me3 was significantly enriched in the outermost zones, compared to more internal regions (Figure 2C, ANOVA p value 8.7×10^−18^).

These results are largely consistent with recent studies describing distinct nuclear patterns of histone modifications in cultured cell lines [43], but extend it by showing that H3-Lys4me2 exhibits preferential colocalization with RNA polymerase II in central nuclear domains, while H3-Lys27me3 is particularly abundant in peripheral domains lacking enrichment in RNA polymerase II, H3-Lys4me2, or H3-Lys9me3.

### Hnf1α-Regulated Gene Loci Display Differential Association with Distinct Subnuclear Domains in *Hnf1a^+/+^* versus *Hnf1a^−/−^* Cells

**Figure 3:**
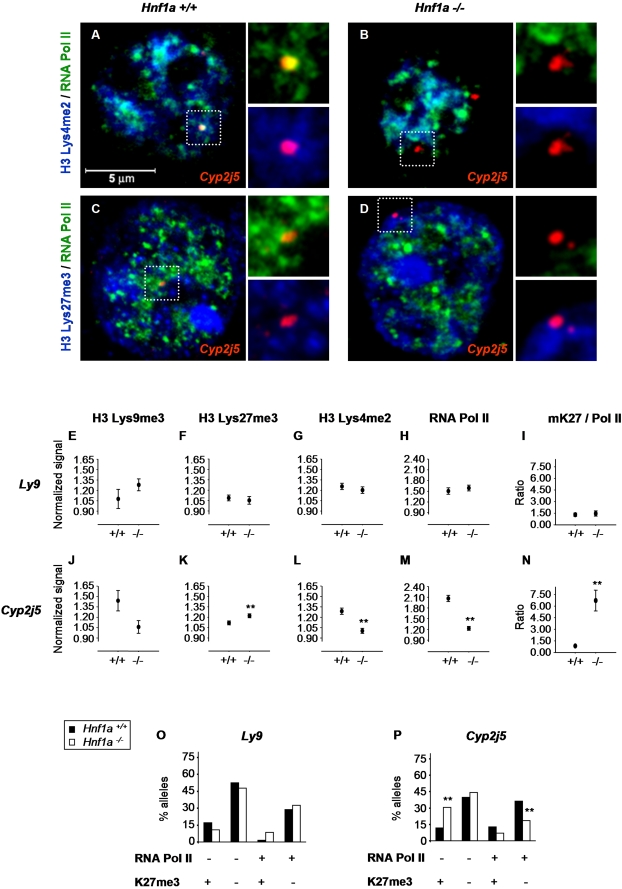
Hnf1α-dependent *Cyp2j5* activity correlates with differential positioning in RNA polymerase II and histone code domains. (A–D) Representative confocal immuno-FISH analysis in *Hnf1a^+/+^* and *Hnf1a^−/−^* hepatocytes of the *Cyp2j5* locus (red) with RNA polymerase II (RNA Pol II, green) and either H3-Lys4me2 (A,B) or H3-Lys27me3 (C,D) (blue). The framed regions containing *Cyp2j5* FISH signals are shown at higher magnification on the right of each panel with omission of blue or green channels (E–N) Quantitative analysis of histone marks and RNA polymerase II in *Cyp2j5* (J–N) and *Ly9* control (E–I) loci in *Hnf1a^+/+^* and *Hnf1a^−/−^* hepatocytes. For each condition, non-thresholded fluorescence intensities of histone marks and RNA polymerase II were measured at 70–200 FISH signals, and each value was divided by its nuclear median intensity in the same channel. The graphs thus depict the average of such *normalized signal* values±SEM, except in I,N which shows mean±SEM of H3-Lys27me3/RNA polymerase II ratios (mK27/Pol II) calculated for each allele. (O–P) Classification of *Cyp2j5* (P) and *Ly9* control (O) alleles into 4 categories according to the simultaneous enrichment (+) or non-enrichment (−) of RNA polymerase II (RNA Pol II) and H3-Lys27me3 (K27me3) in *Hnf1a^+/+^* (black bars) and *Hnf1a^−/−^* (white bars) hepatocytes. Each allele was scored as enriched (+) or non-enriched (−) based on whether or not the signal intensity exceeded the 75th percentile of nuclear signals. Alternate thresholds such as the nuclear median yielded comparably significant results (see text). Results are expressed as % of all alleles for each genotype. *p<0.05 and **p<0.01 relative to *Hnf1a^+/+^* cells using Mann-Whitney or Fisher's exact test as appropriate.

Immunofluorescence analysis indicated that Hnf1α is clearly enriched in H3-Lys4me2- and RNA polymerase II-rich, H3-Lys27me3-poor subnuclear domains, suggesting that there might be a subnuclear compartmentalization of Hnf1α function (Figure S3). We therefore tested if Hnf1α promotes not only changes in site-specific histone modifications, but also in the subnuclear positioning of its targets relative to histone modification domains. To address this question we performed DNA immuno-FISH experiments and non-thresholded images were analyzed to determine the enrichment of defined histone modifications and RNA polymerase II at Hnf1α-dependent loci in control vs. null-mutant nuclei (Figure 3 and Figure S4). Importantly, the compartmentalization of histone marks was conserved after the immuno-FISH procedure, and the spatial patterns of histone modifications were unaltered in *Hnf1a^−/−^* cells (Figure S1A and Figure S5).

In several studies it has been observed that gene silencing is associated with relocation to constitutive heterochromatic domains enriched in satellite repeat sequences [21]–[24],[44]. In mice, H3-Lys9me3 is enriched at pericentromeric regions [11]. In agreement, this was also observed under the conditions used here, where pericentromeric regions clustering at chromocenters were highlighted by TO-PRO-3 staining (Figure S2). However, inactive Hnf1α-targets in mutant hepatocytes (*Cyp2j5*) and islets (*Kif12*) were not positioned in domains that are enriched in H3-Lys9me3 or TO-PRO-3 as compared to wild-type cells (Figure 3J, Figure S2D, and not shown). Furthermore, the distance of *Cyp2j5* to H3-Lys9me3-rich chromocenters, and the frequency with which the two were in contact, did not differ in wild-type vs. null- mutant cells (0.94±0.10 vs. 0.88±0.09 µm, and 10% vs 7.7%, respectively). Thus, Hnf1α-deficiency does not result in repositioning of inactive Hnf1α-targets to pericentromeric heterochromatin clustering at chromocenters enriched in H3-Lys9me3.

In sharp contrast, *Cyp2j5* alleles in activator-deficient cells were positioned in nuclear domains that are relatively enriched in H3-Lys27me3 (Figure 3K). Analogous results were observed for the pancreatic islet Hnf1α-dependent gene *Kif12* (Figure S4I). These observations were specific for Hnf1α-dependent loci because they were not observed in 4 control loci in hepatocytes (*Hnf1b, Ly9*, *Actb* and *Nanog*) (Figure 3F and Figure S6) or one control locus in islet-cells (Figure S4E). Furthermore, silent *Cyp2j5* and *Kif12* loci in *Hnf1a-*deficient cells were located in subnuclear domains with decreased H3-Lys4me2 and RNA polymerase II (Figure 3L,M and Figure S4J,K).

Simultaneous imaging of two protein marks at each locus allowed us to more accurately assess the extent to which loci were differentially positioned in domains enriched in distinct marks. We found that the average ratio of non-thresholded H3-Lys27me3/RNA polymerase II fluorescence signal intensity measured at individual *Cyp2j5* and *Kif12* FISH signals was 8- and 2.3- fold higher in *Hnf1a^−/−^* vs. *Hnf1a^+/+^* cells, respectively, but remained unaltered at control loci (Figure 3I,N; Figure S4H,L; and Figure S6). We also classified alleles according to their presence in domains enriched in histone modifications and RNA polymerase II, using a 75^th^ percentile enrichment criterion, as described above. This analysis showed that Hnf1α-dependent genes *Cyp2j5* and *Kif12* were located in domains selectively enriched in H3-Lys27me3 2.6 and 3.3 times more frequently in *Hnf1a^−/−^* cells compared to *Hnf1a^+/+^* cells, respectively (Figure 3P and Figure S4N). This finding did not reflect just delocalization from RNA polymerase II-rich domains, as *Cyp2j5* and *Kif12* in null-mutant cells were not more frequently in RNA polymerase II-poor/H3-Lys27me3-poor domains (Figure 3P and Figure S4N), nor in RNA polymerase II-poor/H3-Lys9me3-rich or RNA polymerase II-poor/TO-PRO-3-rich domains (data not shown). The results remained significant using the median (50^th^ percentile) of nuclear epitope intensity as an alternate threshold to define epitope enrichment (2.6-fold and 2.2-fold increased presence of *Cyp2j5* and *Kif12* in H3-Lys27me3-rich/RNA polymerase II-poor domains in *Hnf1a^−/−^* vs. *Hnf1a^+/+^* cells, respectively; Fisher's exact test, p<0.01). Differences were again not observed in four control genes using similar criteria (Figure 3O, Figure S4M, and not shown).

In concordance with the preferential nuclear compartmentalization of Hnf1α in RNA polymerase II- and H3-Lys4me2-rich domains (Figure S3), target loci were also preferentially localized in Hnf1α-rich domains in hepatocytes, in contrast to the inactive control locus *Ly9* (Figure S7, and not shown). However, we found no evidence that this preferential localization reflected the existence of an activator-specific subnuclear domain, because an Hnf1α-independent active control gene (*Actb*) exhibited a similar subnuclear compartmentalization with Hnf1α as *Cypj5* (Figure S7).

**Figure 4:**
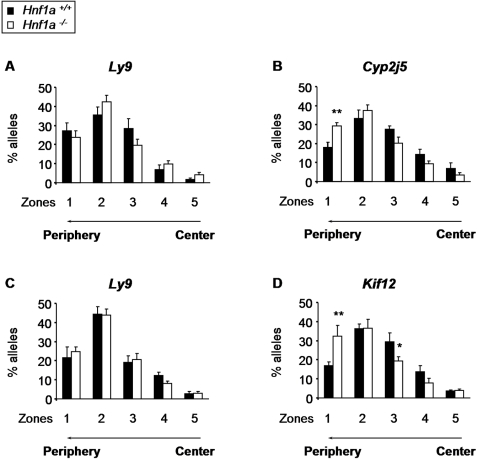
Hnf1α determines the radial nuclear position of its target loci. DNA-FISH erosion analysis of the radial positioning of the *Ly9* control locus (A,C) and the Hnf1α-dependent loci *Cyp2j5* (B) and *Kif12* (D) in *Hnf1a^+/+^* (black bars) and *Hnf1a^−/−^* (white bars) hepatocytes (A,B) and islet-cells (C,D). The nucleus was divided in 5 concentric zones and the percentage of FISH signals present in each zone was determined for each locus and genotype. The graph depicts the mean±SEM. *p<0.05 and **p<0.01 relative to *Hnf1a^+/+^* cells.

We also compared the radial positioning of Hnf1α-dependent loci by erosion analysis in wild-type vs. mutant hepatocytes and islet cells, respectively. In contrast to the unchanged radial positioning of the control locus *Ly9*, significantly increased percentages of *Cyp2j5* and *Kif12* alleles localized in the most peripheral nuclear zone, where H3-Lys27me3 is mostly enriched, in mutant nuclei compared to wild-type (p = 0.002 and p = 0.01, respectively) (Figure 4). Conversely, a significant decrease in the number of *Kif12* loci in mutant cells was observed in the interior shell 3 (p = 0.04) (Figure 4).

Thus, in the presence of Hnf1α its direct target genes *Cyp2j5* and *Kif12* are positioned in more central nuclear domains enriched in RNA polymerase II and H3-Lys4me2, whereas in the absence of Hnf1α inactive targets are positioned in more peripheral, H3-Lys27me3-rich domains. Interestingly, these subnuclear histone modification enrichment patterns parallel those observed locally in Hnf1α-target nucleosomes.

### Spatial Resolution of Hnf1α-Dependent Positioning

**Figure 5:**
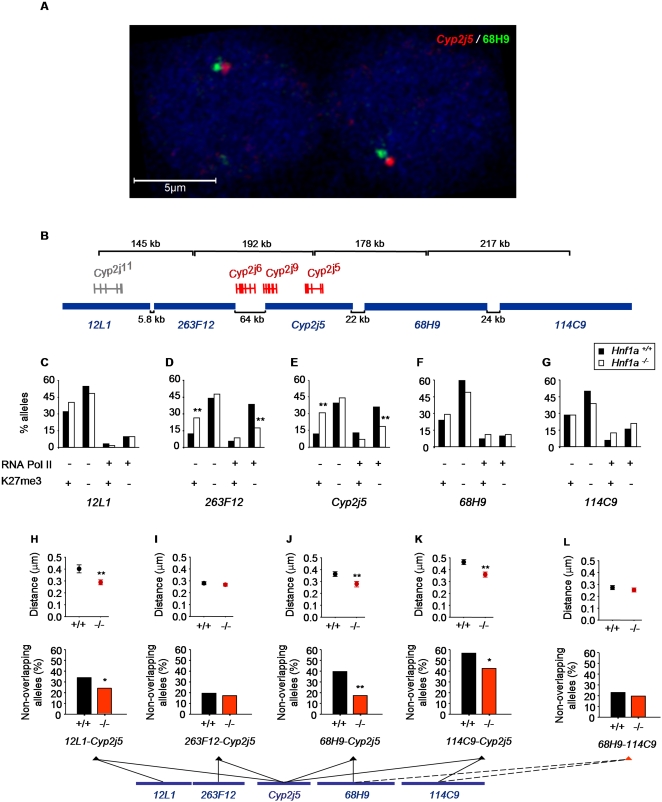
Spatial resolution of Hnf1α-dependent repositioning. (A) Two-color DNA FISH detection of adjacent loci *Cyp2j5* (red) and 68H9 (green) in *Hnf1a^+/+^* hepatocytes. (B) Schematic representation of the relative positions and distances (Kb) of BACs located centromeric (12L1 and 263F12) and telomeric (68H9 and 114C9) to *Cyp2j5*. Hnf1α-dependent genes in the region are drawn schematically in red. Note that no spliced transcript has been mapped to the region encompassed by BACs 68H9 and 114C9. (C–G) Classification of 12L1 (C), 263F12 (D), Cyp2j5 (E), 68H9 (F), and 114C9 (G) alleles into 4 categories according to the simultaneous enrichment (+) or non-enrichment (−) of RNA polymerase II (RNA Pol II) and H3-Lys27me3 (K27me3) in *Hnf1a^+/+^* (black bars) and *Hnf1a^−/−^* (white bars) hepatocytes as described in Figure 3O–P. (H–L) Comparison of distances between the indicated BAC clone FISH signals in *Hnf1a^+/+^* and *Hnf1a^−/−^* hepatocytes. For each comparison, the upper panels show the mean±SEM interlocus (signal center to center) distances in µm, with significance values calculated with the Mann-Whitney test. Lower panels show the percentage of non overlapping alleles, defined as those with signal center distances exceeding 0.4 µm, with significance values assessed with Fisher's exact test. *p<0.05, **p<0.01 relative to *Hnf1a^+/+^* cells. More than 100 nuclei were analyzed in each case.

Altered positioning of Hnf1α targets in null mutant cells could represent a localized activator-dependent phenomenon, or a more global effect of Hnf1α-deficiency on the configuration of nuclear structures. To address the mechanisms involved, we performed two-color DNA FISH using contiguous BAC probes mapping to sites adjacent to the *Cyp2j5* locus (Figure 5). Despite their proximity, signals from adjacent clones could be clearly separated by dual FISH analysis in a substantial number of nuclei (Figure 5A), thus enabling us to test how genomic regions in the vicinity of *Cyp2j5* were positioned relative to subnuclear domains in wild-type and mutant cells. To assist the interpretation of results, we first analyzed the gene content in these regions. We noted that there were two additional Hnf1α-dependent genes immediately centromeric to *Cyp2j5*, while an extensive telomeric region was completely devoid of any experimentally defined spliced transcripts (Figure 5B, Table S2). Parallel ImmunoFISH studies showed that unlike *Cyp2j5,* the adjacent regions 12L1, 68H9, and 114C9 were not differentially distributed with respect to nuclear RNA polymerase II or histone marks domains in *Hnf1a^−/−^* vs. *Hnf1a^+/+^* cells (Figure 5C,F and G). Nonetheless, the region marked by clone 263F12 that is in immediate proximity to the Hnf1α-dependent gene (*Cyp2j6*) did show differential positioning similar to *Cyp2j5* (Figure 5D–E, Table S2). These findings indicated that Hnf1α-dependent positioning of *Cyp2j5* into histone modification/RNA polymerase II subnuclear domains is a locally restricted phenomenon, encompassing a somewhat extended domain of up to 300 Kb containing at least two additional coordinately regulated Hnf1α-dependent genes.

We next sought to determine if Hnf1α-dependent positioning of the *Cyp2j5* locus can be elicited relative to adjacent genomic regions, thus providing reference points that are independent of histone mark and RNA polymerase II spatial distributions. We used two-color DNA FISH to measure the distance of *Cyp2j5* to adjacent loci in wild-type vs. null-mutant cells. We found that the distance between *Cyp2j5* and the two telomeric clones 68H9 and 114C9 was significantly increased in wild-type compared with *Hnf1a^−/−^* hepatocytes (0.36±0.02 vs. 0.28±0.02 µm, and 0.46±0.02 vs 0.36±0.02 µm, respectively, Mann-Whitney test p<0.001) (Figure 5J,K). The distance between *Cyp2j5* and the most proximal centromeric 263F12 region was not affected by *Hnf1a-*deficiency (in keeping with the lack of differences in RNA polymerase II/K27me3 colocalization studies), but for the more distal clone 12L1 it was decreased from 0.40±0.03 µm in wild-type cells to 0.29±0.02 µm in *Hnf1a^−/−^* cells (Mann-Whitney test p<0.001; Figure 5H–I). Accordingly, the percentage of non-overlapping loci, which was systematically defined as those located at >0.4 µm center to center distance, was higher in wild-type vs. null mutant cells for *Cyp2j5*-68H9 (39 vs. 17%, Fisher's exact test p<0.001), *Cyp2j5*-114C9 (57 vs. 42%, p<0.05), and *Cyp2j5-*12L1 (35 vs. 24%, p<0.05) comparisons (Figure 5H–K). In contrast, the distances separating 68H9 and 114C9, which do not contain Hnf1α*-*dependent genes, do not differ between control and null-mutant cells (Figure 5L). Thus, *Cyp2j5* showed altered Hnf1α-dependent positioning relative to neighboring centromeric and telomeric chromosomal regions.

We further assessed Hnf1α-dependent positioning of *Cyp2j5* with respect to its chromosomal territory. We observed that Hnf1α-deficiency did not affect the position of the nearby 114C9 genomic region relative to its chromosomal territory, whereas *Cyp2j5* alleles less frequently extended away from their territory surface in *Hnf1a^−/−^* cells versus wild-type cells (Figure S8). Collectively, these findings reveal the existence of Hnf1α-dependent, spatially restricted positioning of a target locus relative to chromosomal reference landmarks and subnuclear RNA polymerase II/histone modification domains. The analysis of distances between adjacent regions and relative to the chromosome territory furthermore indicates that Hnf1α-dependent positioning involves chromatin decondensation of the *Cyp2j5* locus.

## Discussion

### Genetic Evidence for Activator-Dependent Gene Positioning

We have used a genetic model to show that a transcriptional activator regulates the subnuclear positioning of its direct endogenous targets in primary differentiated cells. We documented Hnf1α-dependent differential gene positioning with respect to: a) subnuclear regions enriched in H3-Lys27me3, H3-Lys4me2, and phosphoserine-5 RNA polymerase II (Figure 3), b) radial nuclear zones (Figure 4), c) genomic regions adjacent to an Hnf1α-dependent gene (Figure 5), and d) chromosomal territories (Figure S8). The analysis of four control loci in *trans* allowed us to conclude that the observed Hnf1α-dependent spatial changes are specific. Experiments comparing the position of an Hnf1α-dependent locus to adjacent chromosomal regions and its chromosomal territory further demonstrated specificity, and revealed that changes were locus-selective and did not reflect broad chromosomal reconfigurations.

Although numerous studies have shown a relationship between gene transcription and subnuclear positioning, several variables that are only indirectly related to gene transcription, such as regional gene density or nucleotide composition, also appear to impact the subnuclear location of genomic regions, independent of their actual transcriptional activity [27]–[29],[45]. Our new findings demonstrate that transactivator-dependent functions are dominant over such variables in the regulation of subnuclear gene positioning.

Earlier reports have linked the function of sequence specific-DNA binding proteins such as Ikaros and NF-E2p18 with the repositioning of endogenous loci [21],[46],[47]. In such examples, repressor-mediated repositioning of silenced loci to pericentromeric compartments was observed during developmentally regulated gene-silencing processes. This clearly represents a different situation compared to the current analysis where gene inactivity results from the sheer lack of an activator and gives rise to a different pattern of subnuclear positioning that does not involve association with chromocenters.

Previous evidence supporting the role of transactivators in gene positioning comes from studies of transgenes. Some of these studies took advantage of a lac repressor-VP16 acidic activation domain fusion protein, which was shown to cause repositioning of targeted multicopy loci away from the nuclear periphery [31],[32]. Another study has analyzed transgenes with intact or mutated transactivator binding sites and showed that intact sites prevent association of transgenes with pericentromeric heterochromatin [48]. The role of transactivators in the positioning of endogenous loci, however, has not been directly assessed. One study showed that the deletion of a 24 Kb endogenous genomic region containing the β-globin locus control region results in gene silencing and increased perinuclear localization of the endogenous locus [49]. These effects were probably due to activator functions because the deleted region contained multiple binding sites for essential transcription factors. Nevertheless, it could not be excluded that structural changes due to deletion of an extended genomic segment also affected nuclear positioning by transactivator independent mechanisms. Our results provide genetic evidence in primary cells that positioning of endogenous genes can be dependent on a single transactivator. Together with previous studies, this suggests that the regulation of the subnuclear location of target gene loci might be a general function of sequence-specific DNA binding transcriptional regulators.

### Gene Positioning Relative to Subnuclear Domains

Earlier studies describing correlations between gene silencing and perinuclear positioning were based on the comparisons of different cell types or developmental stages [19],[20],[50],[51]. Such studies can theoretically be confounded by cell-specific differences in global spatial chromosomal arrangements [39]. It is thus important that peripheral positioning is now elicited in a model where transcriptional inactivity is ascribed to the selective absence of a direct transactivator.

Previous studies have also shown that genes are preferentially transcribed in nuclear subdomains enriched in RNA polymerase II [17],[52],[53]. This has led to models postulating that active loci loop into domains with high local RNA polymerase II concentrations [17],[54]. Our findings confirm that gene activity is associated with localization to phosphoserine-5 RNA polymerase II domains in primary cells, and furthermore demonstrate that association with such domains is linked to the function of a transcriptional activator. Importantly, the new results extend our understanding of this phenomenon by showing that relocation does not only occur with respect to domains enriched in RNA polymerase II, but also involves repositioning amongst compartments that differ in the composition of histone modifications known to be critically involved in transcriptional regulation, and that such domains display distinct radial distributions. Our integrated analysis of a transactivator-deficient model thus suggests that transcription-related gene positioning with respect to RNA polymerase II foci, distinct radial nuclear zones, and domains enriched in specific histone modifications might reflect different experimental measurements of a single biological phenomenon.

### Relationships between Hnf1α-Dependent Locus Specific Chromatin Changes and Subnuclear Positioning

**Figure 6:**
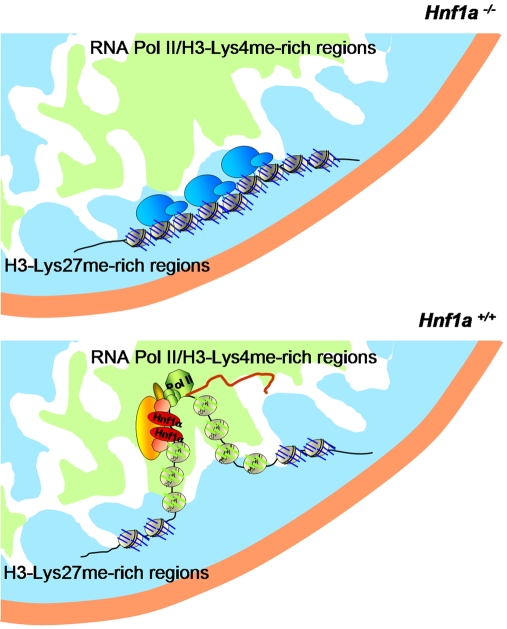
Summary model. *Hnf1a^−/−^.* In the absence of Hnf1α, target nucleosomes exhibit increased trimethylated H3-Lys27, and are more likely to be located in peripheral nuclear domains with condensed chromatin enriched in methylated H3-Lys27. *Hnf1a^+/+^*: In wild-type cells, Hnf1α binding recruits complexes that lead to site-specific histone acetylation, H3-Lys4 methylation, and chromatin remodeling, while preventing trimethylation of H3-Lys27. This chromatin configuration is associated with relocalization to transcriptionally active, more centrally located nuclear subregions enriched in RNA polymerase II and H3-Lys4me2. We propose that activator-dependent local chromatin changes may be instrumental in gene positioning.

Together with previous findings, our data shows that binding of Hnf1α to target loci promotes local histone tail hyperacetylation, methylation of H3-Lys4, and chromatin decondensation, while preventing methylation at H3-Lys27 [38],[40],[41]. H3-Lys27 methylation thus appears to represent a default state, consistent with genetic studies showing that the H3-Lys4-specific methyltransferase Trithorax suppresses default gene silencing mediated by methylated H3-Lys27 [55]. Concomitant with local chromatin changes, Hnf1α binding also causes the recruitment of targets to predominantly central subdomains that are enriched in phosphoserine-5 RNA polymerase II and concordant histone modifications (see model in Figure 6).

How Hnf1α controls subnuclear positioning of its targets remains to be clarified. Treatment with the RNA polymerase II inhibitors α-amanitin and 5,6-dichlorobenzimidazole riboside (DRB) does not alter the preferential positioning of *Kif12* in H3-Lys4me-rich/H3-Lys27me-poor domains in a β-cell line with normal Hnf1α expression (Figure S9). This suggests that *Kif12* compartmentalization is not solely dependent on ongoing transcriptional activity *per se,* and points to the involvement of other activator-dependent functions.

Changes in local chromatin structure represent another potential mechanism. Our results showed that Hnf1α regulates not only local chromatin decompaction, but also the decondensation of the *Cyp2j5* locus that is reflected by changes in distances between adjacent loci and relative to chromosomal territories. Our findings also show that the histone modification enrichment pattern of Hnf1α-dependent genes in nuclear domains with which they associate coincides with the local post-translational histone modification profile. This raises the possibility that histone modifications may be partly instrumental in gene positioning. Although similar measurements of locus positioning relative to histone modification domains have not been carried out before, two studies previously showed that treatment with histone deacetylase inhibitors causes repositioning of inactive genes away from the nuclear periphery [20],[56]. Local histone modifications could affect compartmentalization of gene loci by regulating interactions with the nuclear lamina [56] and could also affect mobility, since acetylated histones have been previously shown to increase chromatin fiber flexibility [57]. Taken together, these findings support the proposal that local Hnf1α-dependent chromatin decompaction and histone modifications might result in augmented mobility and loop formation, thus increasing the likelihood of accessing and establishing dynamic interactions with components of transcriptionally active nuclear regions (Figure 6). Local activator-dependent changes in chromatin structure may thus play a role in regulating the spatial organization of the genome.

Emerging evidence indicates that gene transcription is an integrated process involving multiple levels of regulation [2],[3]. The data presented here link the *in vivo* function of an activator to different levels of regulation, namely the binding to specific target sequences, the local modification of target chromatin, and the positioning of targets in distinct subnuclear domains. This demonstration is provided in a genetic model of human diabetes, indicating cellular defects at multiple regulatory levels in a human transcriptional disease. Thus, our findings provide not only new insights into the complexity of trans-activator functions and transcriptional regulation, but are also important for understanding mechanisms underlying human disease.

## Material and Methods

### Cell Preparation

Hepatocytes and pancreatic islets were isolated from 4–6 week-old *Hnf1a^+/+^* and *Hnf1a^−/−^* mice [36] by local perfusion of the organ with collagenase for digestion and subsequent isolation of the cells as described [34],[40]. For immnostaining and FISH studies, after isolation islets were gently dissociated for 2 min in pre-warmed trypsin solution. Cells were processed for chromatin, RNA, immunofluorescence, and FISH analysis immediately after isolation.

### RNA Extraction and RT-PCR

RNA isolation, reverse transcription and PCR were carried out as described [34].

### General DNAse I Sensitive Assay

Isolated hepatocytes (30–40.10^6^) were resuspended in 10 mL NI buffer (15 mM Tris-HCl pH7.5, 300 mM sucrose, 15 mM NaCl, 60 mM KCl, 4 mM MgCl2 and 0.5 mM DTT), and 10 mL of NI buffer supplemented with 1% NP40 was added for 10 min incubation in ice. Nuclei were collected at 500×g for 3 min, washed in 4 mL NI buffer, resuspended in a final volume of 700 µL NI buffer and distributed in 100 µL aliquots for DNAse I digestion. To each suspension of 100 µL NI buffer, either 0, 10, 20, 30, 40, 50 or 80 µg DNAse I was added for 10 min on ice. The reaction was stopped with Nuclei Lysis Solution from Wizard Genomic DNA purification kit (Promega), and DNA was extracted as indicated by the manufacturer. DNAs were resuspended in 20 µL DNA rehydration solution and 1 µL was used for PCR amplification. Oligonucleotides are presented in Table S1.

### Chromatin Immunoprecipitation

Approximately 2.10^6^ isolated hepatocytes or MIN6 beta-cells were used per immunoprecipitation as described [40],[58], with modifications. Immunoprecipitations were carried out overnight at 4°C with 7.5 µg rabbit anti-HNF1 (H-205) (Santa Cruz, sc8986), 2 µg rabbit anti-H3-Lys4me2 (Upstate, 07-030), 2 µg mouse anti-H3-Lys27me2,3 [59] (D. Reinberg, University of Medicine and Dentistry of New Jersey), 10 µg rabbit anti-H3-Lys27me3 [11] (T. Jenuwein, The Vienna Biocenter and Upstate, 07-449) or 20 µg rabbit anti-H3-Lys9me3 [11] (T. Jenuwein and Upstate, 07-442). For anti-H3-Lys4me2 and H3-Lys27me2,3, 1% Triton was added to the antibody binding solution. For anti-H3-Lys27me2,3, 3 µg rabbit anti-mouse IgG (Sigma) was added for a further 3 hr incubation at 4°C. Immune complexes were collected by adsorption to protein A+G Sepharose (Amerhsam). Beads were washed and eluted as described, except for the anti-H3-Lys9me3 immunoprecipitation that was washed with 250 mM NaCl. Purified immunoprecipitated DNA was analyzed in duplicate by SYBR green real-time PCR, and compared to a standard curve generated with serial dilutions of input chromatin DNA. Oligonucleotides are shown in Table S1.

### Immunofluorescence

5.10^5^ isolated hepatocytes or islet-cells were lightly cytospun and fixed at room temperature for 5 min in freshly prepared 4% paraformaldehyde. In control experiments immunostaining was carried out as described [60] except that antibody retrieval was not employed. Primary antibodies were used with the following dilutions: rabbit anti-H3 (Abcam, Ab1791) (1/500), rabbit anti H3-Lys4me2 (Upstate, 07-030) (1/500), rabbit anti H3-Lys9me3 [11] (T. Jenuwein, and Upstate, 07-442) (1/500), rabbit anti H3-Lys9me2 [11] (T. Jenuwein, and Upstate, 07-442) (1/500), mouse anti-H3-Lys27me3 (Abcam, Ab 6002) (1/50), rabbit anti H3-Lys27me3 [11] (T. Jenuwein and Upstate, 07-449) (1/500), rabbit anti H3-Lys27me2 [11] (T. Jenuwein) (1/500), rabbit anti H3-Lys27me1 (Upstate, 07-448) (1/500), mouse anti-phospho serine 5 RNA polymerase II CTD4H8 (Abcam, Ab 5408) (1/1000), goat anti-Lamin A/C N18 (Santa Cruz) (1/200), mouse anti-HNF1α (Transduction Laboratories, H69220) (1/50), and rabbit anti-HNF1 (H-205) (Santa Cruz, sc8986) (1/100). The specificity of methylated H3-Lys27 stainings was verified by co-staining with two different highly specific antibodies directed against the same epitope and using alternate fixation (methanol at −20°C for 10 min). The specificity of anti-Hnf1α staining patterns was verified using *Hnf1a^−/−^* cells and alternate fixation procedures (Figure S3). Secondary donkey antibodies anti-mouse Cy2, anti-mouse Cy3, anti-mouse IgM Cy3, anti-goat Cy2, anti-rabbit Cy5 and anti-rabbit Cy3 were from Jackson ImmunoResearch, and used at 1/200. Nuclear DNA was counterstained with TO-PRO-3 (1/50,000).

### DNA Immuno-FISH

We used purified BAC DNAs (Table S2) for labeling with Dig-nick translation or BioNick kits (Roche). Immuno-FISH was based on modifications of the protocol described by Brown et al [21]. Cells were immunostained essentially as described above except that nuclei were fixed in 4% paraformaldehyde for 15 min and heated in a microwave in 10 mM citrate buffer, pH 6, for 5 min before permeabilization. Immunostained nuclei were then post-fixed in 4% paraformaldehyde for 15 min, denatured in NaOH 0.1M in PBS, pH 13 for 110 sec, and washed in cold PBS and 2× SSC. One µL digoxigenin-labeled probe in 14 µL hybridization buffer (50% formamide, 2× SSC, 125 µg/mL Cot-1 and 10% dextran sulphate) and 1 µg mouse Cot-1 (Invitrogen) were denatured for 5 min at 90°C. Probes were hybridized overnight at 37°C, and sequentially washed in 2×SSC, 1×SSC, PBS-triton 0.2%, and PBS for 5 min at room temp. Slides were then sequentially incubated with Sheep anti-digoxigenin antibody (Roche) (1/300) for 3 h, and donkey anti-sheep Cy3 antibody (Jackson Immunoresearch) (1/200) for 2 h at room temp., with washes after each step. Cells were mounted with ProLong Antifade (Amersham).

### Two-Color DNA FISH

Cells were fixed in 4% paraformaldehyde for 15 min at room temperature, washed in PBS and permeabilized for 30 min in PBS-0.5% Triton X-100. Cells were then heated in 10 mM citrate buffer, pH 6 for 5 min and post-fixed in 4% paraformaldehyde, washed in PBS, and incubated in 2× SSC. One µL digoxigenin-labeled probe and one µL biotin-labeled probe were added to 14 µL hybridization buffer as described above. Both probes and cells were simultaneously heated at 90°c for 5 min to denature DNA, and hybridized and washed essentially as in the immuno-FISH protocol. The digoxigenin-labeled probe was detected as in the immuno-FISH procedure, and the biotin-labeled probe was detected with AF488-streptavidin (Molecular Probes)(1/500). Cells were washed and counterstained with TO-PRO-3 (Molecular Probes)(1/50,000) and mounted with ProLong Antifade (Amersham).

### Chromosome Painting

Cells were fixed and permeabilized as described above. After permeabilization, hepatocytes were incubated with 100 µg/mL RNase A at 37°C for 30 min. Nuclei were then denatured at 74°C in 2×SSC-70% formamide for 3 min followed by 1 min in 2×SSC-50% formamide. Ten µL of chromosome 4 biotin-labeled probe (Cambio) was denatured at 70°C for 10 min in the supplied buffer (Cambio) and pre-annealed for 20 min at RT. Subsequently 1 µL of either *Cyp2j5* or *114C9* digoxigenin-labeled probe denatured at 90°C for 5 min was added for overnight hybridization at 37°C. After sequential washes of 5 min at 45°C in 2×SSC-50% formaldehyde, 1×SSC, PBS-0.2% Triton and PBS, biotin and digoxigenin-labeled probes were detected and processed for confocal image acquisition as described above.

### Image Collection

Confocal images for each fluorochrome were acquired sequentially at room temperature with a Leica TCS SL laser scanning confocal spectral microscope, using a 63× oil immersion objective lens (NA 1.32). Focal Check Fluorescent microspheres (Molecular Probes) were used before image capture to align laser lines. Non-saturated, unprocessed images were further analyzed with ImageJ. Contrast-stretch and gamma adjustments were made using Photoshop (Adobe) only for display.

### Colocalization Analysis

This analysis was carried out to determine colocalization between the most intense nuclear signals of each epitope. Ten to twenty nuclei were assessed in each double immunofluorescence experiment, and pixels with values exceeding the 75th percentile in each channel were selected for further analysis. The rationale for this threshold is that nuclear RNA polymerase II and H3-Lys27me3 signal intensities do not adhere to a normal distribution, and the 75th percentile enabled separation of visually evident RNA polymerase II and H3-Lys27me3-enriched domains from most remaining nuclear signals. Signals filtered in this manner were used to calculate Manders' coefficient of colocalization using the appropriate ImageJ plug-in (Wayne Rasband and Tony Collins, www.uhnresearch.ca/wcif). Manders' coefficient calculates, for each channel, the proportion of colocalizing pixels respect to the summed up intensities of all pixels in the nucleus. Comparable results were obtained by subtracting pixels lower than the 50th percentile in each channel, and then applying a modified Mander's coefficient using the Colocalization threshold ImageJ plug-in, that first calculates an automated threshold (% colocalized pixels above threshold: RNA polymerase II vs. H3-Lys9me3: 4%. RNA polymerase II vs. H3-Lys27me3: 19%. RNA polymerase II vs. H3-Lys4me2: 73%. H3-Lys27me3 vs. H3-Lys4me2: 22.5%. H3-Lys27me3 vs. H3-Lys9me3: 31%).

### Erosion Analysis

Erosion analyses were performed with single light optical sections and mid-nuclear planes were analyzed. Radial positioning of FISH signals was analyzed as described [20]. With respect to immunostaining data erosion analyses were performed as follows. Based on the DNA counterstaining signal, the nuclear plane was subdivided into five concentric zones, each having a thickness of 20% of the nuclear radius. The numbers of pixels in each zone were counted and the grey value of each pixel was determined separately for each channel. Each grey value (I) was multiplied by its frequency N (I×N) and the sum of all values obtained for a given zone was determined (Σ I×N). The result obtained for each zone was divided by the sum of results obtained for all nuclear zones in order to determine the percentage of total nuclear fluorescence intensity in each nuclear zone. The values obtained were normalized to the relative nuclear areas occupied by the different zones. Thus a value of 1 was obtained if the percentage of the total nuclear fluorescence intensity corresponded to the percentage of the total nuclear area occupied by a given zone. It should be noted that this procedure did not involve any thresholding.

### Immuno-FISH Image Analysis

For each condition, typically 100 alleles (range 70–200) from at least two independent experiments were analyzed blindly in unprocessed images to quantify signal intensities of histone marks and RNA polymerase II. In H3-Lys27me3 experiments Barr bodies were avoided. The 9-pixel area containing the brightest and most central pixels of each FISH signal was selected by inspection of single color images, and the average signal for each channel in this area was obtained using RGB Measure (ImageJ). Each non-thresholded immunofluorescence signal at a FISH-detected locus was divided by the median value of the entire nucleus in the same cell to correct for cell to cell and inter-assay technical variability. The mean intensity for each channel was also calculated from a broad cytoplasmic area in every stack and used to subtract non-specific background from both FISH and nuclear signals. This background value was similar to the non-specific nuclear signal elicited in control Immuno-FISH experiments in which primary antibodies were omitted. The resulting value was referred to as *normalized signal* in Figure 3 and Figure S4).

To classify alleles according to the presence or absence of enrichment in either RNA polymerase II or a histone mark, we used the 75th percentile of nuclear pixel intensities in each channel as the threshold, as described above. The results presented here remained statistically significant with alternate thresholds to define enrichment, such as the nuclear median (see results).

### Two-Color DNA FISH Image Analysis

Approximately 100 allele pairs were analyzed blindly in unprocessed images to quantify the distance (in µm) between the center of adjacent FISH signals defined by the 9-pixel square area containing the brightest and most central pixels, essentially as described for immuno-FISH analysis. Non-overlapping loci were defined as those with signal center distances exceeding 0.4 µm, thus providing a uniform criteria that is not affected by variable FISH signal intensities and shapes.

### Chromosome Painting Image Analysis

After identification of locus-specific probes in the same z plane as its chromosome territory, the image background of the chromosome territory was blindly modified until a clear visualization of the territory edge was obtained. The distance (in µm) between the center of the locus-specific FISH signal and the nearest chromosome surface was then measured as described for the two-color DNA FISH analysis. One hundred alleles from each genotype were classified as being located either within a territory and >0.4 µm from the edge, outside and >0.4 µm from the edge, or in contact if they were <0.4 µm from the edge.

### Statistical Analysis

A two-tailed Student's *t*-test was used for comparison of ChIP values. Mann-Whitney test was used for comparisons of immuno-FISH and allele distance values, which did not adhere to a normal distribution. ANOVA was used for erosion analysis. Fisher's exact test was used for comparison of qualitative two-color DNA FISH, chromosomal territory, and immuno-FISH results.

## Supporting Information

Figure S1 Immunofluorescence analysis of H3-Lys27me3 in hepatocytes. (A) Dual immunofluorescence analysis of RNA polymerase II (green) and H3-Lys27me3 (red) in hepatocytes after the immuno-FISH procedure showing that this process does not alter staining patterns. (B–F) Dual immunofluorescence analysis of hepatocytes with anti-H3-Lys27me3 (green) and either an alternate anti-Lys27me3 antibody (B), or anti-H3-Lys4me2 (C), anti-Lys9me2 (D), anti-Lys27me2 (E) or anti-Lys9me3 (F) antibodies (red). Colocalization analyses are depicted as Venn diagrams on the right side of immunolocalization images and were performed as described in the legend of Figure 2.
(7.62 MB TIF)

Figure S2 Analysis of the correlation between TO-PRO-3 density and histone modification patterns and gene positioning. (A–C) Immunofluorescence analysis of H3-Lys4me2 (A), H3 Lys9me3 (B), and H3-Lys27me3 (C) enrichment (red) compared with the DNA marker TO-PRO-3 (blue). Colocalization was analyzed and depicted with Venn diagrams as described in Figure 2. (D). Quantitative analysis of H3-Lys27me3 and TO-PRO-3 enrichment in *Cyp2j5* loci in *Hnf1a* ^+/+^ (+/+) and *Hnf1a^−/−^* (−/−) hepatocytes. Non-thresholded signal intensities of methylated histone marks or TO-PRO-3 were measured at 70-200 FISH alleles. To correct for cell to cell variability each value was divided by its nuclear median value, and is referred to as the *normalized signal* in the graphs. The graphs depict mean±SEM values. **P<0.01 relative to *Hnf1a* ^+/+^ cells.
(4.07 MB TIF)

Figure S3 Subnuclear distribution of Hnf1α in histone code and RNA polymerase II domains. (A,B) Dual confocal immunofluorescence analysis of Hnf1α (red) and H3-Lys4me2 (A) or H3-Lys27me3 (B) (green) in interphase hepatocyte nuclei. (C–E) Dual immunofluorescence analysis of Hnf1α (red) and RNA polymerase II (green) in control hepatocytes fixed with 4% paraformaldehyde (C) or methanol (D) and in *Hnf1a^−/−^* hepatocytes fixed with 4% paraformaldehyde (E). Colocalization analysis was performed and Venn diagrams were arranged as described in the legend of Figure 2.
(4.12 MB TIF)

Figure S4 Hnf1α-dependent *Kif12* activity in islet-cells correlates with differential positioning in RNA polymerase II and histone code domains. (A–D) Representative confocal immuno-FISH analysis in *Hnf1a* ^+/+^ and *Hnf1a^−/−^* islet-cells of the *Kif12* locus (red) with RNA polymerase II (RNA Pol II, green) and either H3-Lys4me2 (A,B) or H3-Lys27me3 (C,D) (blue). The framed regions containing FISH signals of Kif12 are shown at higher magnification on the right of each panel with omission of only blue or green channels. (E–L) Quantitative analysis of histone marks and RNA polymerase II in *Kif12* (I–L) and control (*Ly9,* E–H) loci in *Hnf1a* ^+/+^ and *Hnf1a^−/−^* islets. For each condition, non-thresholded signal intensities were measured at 100–200 FISH signals and each value was divided by the nuclear median intensity in the same channel. The graphs thus depict the average of such *normalized signal* values±SEM, except in H,L which shows mean±SEM of H3-Lys27me3/RNA polymerase II ratios (mK27/Pol II). (M,N) Classification of alleles from *Ly9* control (M) and *Kif12* (N) in 4 categories according to the simultaneous enrichment (+) or non-enrichment (−) of RNA polymerase II (RNA Pol II) and H3-Lys27me3 (K27me3) in *Hnf1a* ^+/+^ (black bars) and *Hnf1a* ^−/−^ (white bars) islets as described in Figure 3O-P. Results are expressed as % of alleles for each genotype. **p<0.01 relative to *Hnf1a* ^+/+^ cells using Mann-Whitney or Fisher's exact test as appropriate.
(3.89 MB TIF)

Figure S5 Histone modification/RNA Polymerase II colocalization and radial distribution patterns are similar in *Hnf1a^+/+^* and *Hnf1a^−/−^* hepatocytes. (A–C) Venn diagrams showing the colocalization of H3-Lys4me2 (A), H3-Lys9me3 (B) and H3-Lys27me3 (C) with RNA polymerase II in interphase *Hnf1a^+/+^* and *Hnf1a^−/−^* hepatocytes. The mean±SEM percentage of colocalizing pixels of 20 nuclei is shown below. (D–G). Erosion analysis of the nuclear distribution of RNA polymerase II (D, E), and H3-Lys27me3 (F,G) in interphase *Hnf1a^+/+^* and *Hnf1a^−/−^* hepatocytes. Erosion analyses were performed as described in the legend of Figure 2.
(0.61 MB TIF)

Figure S6 Quantitative analysis of histone marks and RNA polymerase II at control genes in *Hnf1a* ^+/+^ and *Hnf1a^−/−^* hepatocytes by DNA immuno-FISH. Non-thresholded signal intensities were measured at 100-200 FISH signals and corrected by the nuclear median value (*normalized signal*) exactly as described in the legend of Figure 3. The graphs depict mean±SEM values. *P<0.05 relative to *Hnf1a* ^+/+^ cells.
(0.35 MB TIF)

Figure S7 Active Hnf1α-dependent and independent loci preferentially localize in Hnf1α-rich domains. (A) Quantitative analysis of Hnf1α immunofluorescence signal intensity at the Hnf1α-target locus *Cyp2j5,* at a non Hnf1α-target active control gene (*Actb*), and at an inactive locus (*Ly9)* in wild-type hepatocytes. Immunofluorescence signals were normalized as described in Figure 3. (B) Percentage of alleles located in RNA polymerase II-rich domains that are also located in Hnf1α enriched domains in wild-type hepatocytes. Immunofluorescence signals were normalized and categorized essentially as described in Figure 3. *P<0.05 and **P<0.01 relative to *Ly9*.
(0.22 MB TIF)

Figure S8 Hnf1α-deficiency causes altered positioning of *Cyp2j5* relative to its chromosome territory. (A–C) Representative confocal images of *Cyp2j5* DNA FISH (red) and chromosome 4 paint (green) in hepatocytes counterstained with TO-PRO-3 (blue) showing alleles that are classified as being either in, out or in contact of the chromosome territory. More details on the criteria for classification are described in methods. Higher magnifications of *Cyp2j5* FISH signals are shown in the upper right panels. (D–E) Percentage of *Cyp2j5* (D) and 114C9 (E) alleles that are located in, out, or in contact with the chromosome 4 territory in *Hnf1a* ^+/+^ (black bars) and *Hnf1a* ^−/−^ (white bars) hepatocytes. Significance values for the comparison of allele distributions between *Hnf1a* ^+/+^ and *Hnf1a^−^* ^/−^ hepatocytes were obtained by Fisher's exact test.
(1.76 MB TIF)

Figure S9 Inhibition of transcriptional activity does not modify *Kif12* positioning in RNA polymerase II and histone code domains. Graphs show immuno-DNA FISH quantitation of H3-Lys4me2, H3-Lys27me3, and RNA polymerase II fluorescence at the *Kif12* locus in MIN6 beta-cells treated for 4 hr with RNA polymerase II inhibitors α-amanitin (50 µg/mL) or 5,6-dichloro-1-beta-D-ribobenzimidazole (DRB) (20 µg/mL) vs. non-treated cells. Non-thresholded fluorescence signal intensities of histone marks and RNA polymerase II at 160-200 FISH signals were divided by the nuclear median intensity in the same channel, and are referred to as *normalized signal* in the graphs, essentially as described in Figure 3. The graphs depict mean±SEM values. Note that in the α-amanitin-treated cells RNA polymerase II fluorescence is not measured because this treatment results in marked reduction of RNA polymerase II foci.
(0.24 MB TIF)

Table S1 Oligonucleotide sequences used in chromatin immunoprecipitation analysis.
(0.03 MB DOC)

Table S2 Bacterial artificial chromosomes used in this study. * Gene expression is represented as −, +, or ++ based on qualitative judgment of expression (not expressed, relative low expression, or relative high expression, respectively). ND: not determined. For genes marked in bold blue this information has been obtained experimentally by reverse transcription PCR or microarray expression analysis, while for genes marked in black this information has been collected from the Unigene EST Expression profile viewer (www.ncbi.nlm.nih.gov/entrez). ^#^Cyp2j6 locus is immediately adjacent but not included in the BAC clone 263F12. Note that Hnf1β mRNA is mildly increased in Hnf1a^−/−^ hepatocytes.
(0.07 MB DOC)

## References

[1] Kadonaga JT (2004). Regulation of RNA polymerase II transcription by sequence-specific DNA binding factors.. Cell.

[2] Kosak ST, Groudine M (2004). Form follows function: the genomic organization of cellular differentiation.. Genes Dev.

[3] van Driel R, Fransz PF, Verschure PJ (2003). The eukaryotic genome: a system regulated at different hierarchical levels.. J Cell Sci.

[4] Jenuwein T, Allis CD (2001). Translating the histone code.. Science.

[5] Schubeler D, MacAlpine DM, Scalzo D, Wirbelauer C, Kooperberg C (2004). The histone modification pattern of active genes revealed through genome-wide chromatin analysis of a higher eukaryote.. Genes Dev.

[6] Strahl BD, Ohba R, Cook RG, Allis CD (1999). Methylation of histone H3 at lysine 4 is highly conserved and correlates with transcriptionally active nuclei in Tetrahymena.. Proc Natl Acad Sci USA.

[7] Cao R, Wang LJ, Wang HB, Xia L, Erdjument-Bromage H (2002). Role of histone H3 lysine 27 methylation in polycomb-group silencing.. Science.

[8] Mager J, Montgomery ND, de Villena FPM, Magnuson T (2003). Genome imprinting regulated by the mouse Polycomb group protein Eed.. Nature Genet.

[9] Martens JHA, O'Sullivan RJ, Braunschweig U, Opravil S, Radolf M (2005). The profile of repeat-associated histone lysine methylation states in the mouse epigenome.. EMBO J.

[10] Plath K, Fang J, Mlynarczyk-Evans SK, Cao R, Worringer KA (2003). Role of histone H3 lysine 27 methylation in X inactivation.. Science.

[11] Peters AHFM, Kubicek S, Mechtler K, O'Sullivan RJ, Derijck AAHA (2003). Partitioning and plasticity of repressive histone methylation states in mammalian chromatin.. Mol Cell.

[12] Fraser P, Bickmore W (2007). Nuclear organization of the genome and the potential for gene regulation.. Nature.

[13] Cremer T, Cremer C (2001). Chromosome territories, nuclear architecture and gene regulation in mammalian cells.. Nature Rev Genet.

[14] Chambeyron S, Bickmore WA (2004). Chromatin decondensation and nuclear reorganization of the HoxB locus upon induction of transcription.. Genes Dev.

[15] Volpi EV, Chevret E, Jones T, Vatcheva R, Williamson J (2000). Large-scale chromatin organization of the major histocompatibility complex and other regions of human chromosome 6 and its response to interferon in interphase nuclei.. J Cell Sci.

[16] Branco MR, Pombo A (2006). Intermingling of chromosome territories in interphase suggests role in translocations and transcription-dependent associations.. Plos Biol.

[17] Osborne CS, Chakalova L, Brown KE, Carter D, Horton A (2004). Active genes dynamically colocalize to shared sites of ongoing transcription.. Nature Genet.

[18] Wansink DG, Schul W, Vanderkraan I, Vansteensel B, Vandriel R, Dejong L (1993). Fluorescent Labeling of Nascent Rna Reveals Transcription by Rna Polymerase-Ii in Domains Scattered Throughout the Nucleus.. J Cell Biol.

[19] Kosak ST, Skok JA, Medina KL, Riblet R, Le Beau MM (2002). Subnuclear compartmentalization of immunoglobulin loci during lymphocyte development.. Science.

[20] Zink D, Amaral MD, Englmann A, Lang S, Clarke LA (2004). Transcription-dependent spatial arrangements of CFTR and adjacent genes in human cell nuclei.. J Cell Biol.

[21] Brown KE, Guest SS, Smale ST, Hahm K, Merkenschlager M, Fisher AG (1997). Association of transcriptionally silent genes with Ikaros complexes at centromeric heterochromatin.. Cell.

[22] Brown KE, Amoils S, Horn JM, Buckle VJ, Higgs DR (2001). Expression of alpha- and beta-globin genes occurs within different nuclear domains in haemopoietic cells.. Nature Cell Biol.

[23] Dernburg AF, Broman KW, Fung JC, Marshall WF, Philips J (1996). Perturbation of nuclear architecture by long-distance chromosome interactions.. Cell.

[24] Merkenschlager M, Amoils S, Roldan E, Rahemtulla A, O'Connor E (2004). Centromeric repositioning of coreceptor loci predicts their stable silencing and the CD4/CD8 lineage choice.. Journal of Experimental Medicine.

[25] Reddy KL, Zullo JM, Bertolino E, Singh H (2008). Transcriptional repression mediated by repositioning of genes to the nuclear lamina.. Nature.

[26] Kumaran RI, Spector DL (2008). A genetic locus targeted to the nuclear periphery in living cells maintains its transcriptional competence.. J Cell Biol.

[27] Gilbert N, Boyle S, Fiegler H, Woodfine K, Carter NP, Bickmore WA (2004). Chromatin architecture of the human genome: Gene-rich domains are enriched in open chromatin fibers.. Cell.

[28] Kupper K, Kolbl A, Biener D, Dittrich S, von Hase J (2007). Radial chromatin positioning is shaped by local gene density, not by gene expression.. Chromosoma.

[29] Mahy NL, Perry PE, Bickmore WA (2002). Gene density and transcription influence the localization of chromatin outside of chromosome territories detectable by FISH.. J Cell Biol.

[30] Sadoni N, Langer S, Fauth C, Bernardi G, Cremer T (1999). Nuclear organization of mammalian genomes. Polar chromosome territories build up functionally distinct higher order compartments.. J Cell Biol.

[31] Chuang CH, Carpenter AE, Fuchsova B, Johnson T, de Lanerolle P, Belmont AS (2006). Long-range directional movement of an interphase chromosome site.. Curr Biol.

[32] Tumbar T, Belmont AS (2001). Interphase movements of a DNA chromosome region modulated by VP16 transcriptional activator.. Nature Cell Biol.

[33] Yamagata K, Oda N, Kaisaki PJ, Menzel S, Furuta H (1996). Mutations in the hepatocyte nuclear factor-1 alpha gene in maturity-onset diabetes of the young (MODY3).. Nature.

[34] Boj SF, Parrizas M, Maestro MA, Ferrer J (2001). A transcription factor regulatory circuit in differentiated pancreatic cells.. Proc Natl Acad Sci USA.

[35] Servitja JM, Ferrer J (2004). Transcriptional networks controlling pancreatic development and beta cell function.. Diabetologia.

[36] Lee YH, Sauer B, Gonzalez FJ (1998). Laron dwarfism and non-insulin-dependent diabetes mellitus in the Hnf-1 alpha knockout mouse.. Mol Cell Biol.

[37] Pontoglio M, Barra J, Hadchouel M, Doyen A, Kress C (1996). Hepatocyte nuclear factor 1 inactivation results in hepatic dysfunction, phenylketonuria, and renal Fanconi syndrome.. Cell.

[38] Pontoglio M, Faust DM, Doyen A, Yaniv M, Weiss MC (1997). Hepatocyte nuclear factor 1 alpha gene inactivation impairs chromatin remodeling and demethylation of the phenylalanine hydroxylase gene.. Mol Cell Biol.

[39] Bolzer A, Kreth G, Solovei I, Koehler D, Saracoglu K (2005). Three-dimensional maps of all chromosomes in human male fibroblast nuclei and prometaphase rosettes.. Plos Biol.

[40] Parrizas M, Maestro MA, Boj SF, Paniagua A, Casamitjana R (2001). Hepatic nuclear factor 1-alpha directs nucleosomal hyperacetylation to its tissue-specific transcriptional targets.. Mol Cell Biol.

[41] Soutoglou E, Viollet B, Vaxillaire M, Yaniv M, Pontoglio M, Talianidis I (2001). Transcription factor-dependent regulation of CBP and P/CAF histone acetyltransferase activity.. EMBO J.

[42] Phatnani HP, Greenleaf AL (2006). Phosphorylation and functions of the RNA polymerase II CTD.. Genes Dev.

[43] Zinner R, Albiez H, Walter J, Peters AHFM, Cremer T, Cremer M (2006). Histone lysine methylation patterns in human cell types are arranged in distinct three-dimensional nuclear zones.. Histochem and Cell Biol.

[44] Brown KE, Baxter J, Graf D, Merkenschlager M, Fisher AG (1999). Dynamic repositioning of genes in the nucleus of lymphocytes preparing for cell division.. Mol Cell.

[45] Zink D (2006). The temporal program of DNA replication: new insights into old questions.. Chromosoma.

[46] Cobb BS, Morales-Alcelay S, Kleiger G, Brown KE, Fisher AG, Smale ST (2000). Targeting of Ikaros to pericentromeric heterochromatin by direct DNA binding.. Genes Dev.

[47] Su RC, Brown KE, Saaber S, Fisher AG, Merkenschlager M, Smale ST (2004). Dynamic assembly of silent chromatin during thymocyte maturation.. Nature Genet.

[48] Francastel C, Magis W, Groudine M (2001). Nuclear relocation of a transactivator subunit precedes target gene activation.. Proc Natl Acad Sci USA.

[49] Ragoczy T, Bender MA, Telling A, Byron R, Groudine M (2006). The locus control region is required for association of the murine beta-globin locus with engaged transcription factories during erythroid maturation.. Genes Dev.

[50] Gasser SM (2001). Positions of potential: Nuclear organization and gene expression.. Cell.

[51] Williams RRE, Azuara V, Perry P, Sauer S, Dvorkina M (2006). Neural induction promotes large-scale chromatin reorganisation of the Mash1 locus.. J Cell Sci.

[52] Grande MA, van der Kraan I, de Jong L, van Driel R (1997). Nuclear distribution of transcription factors in relation to sites of transcription and RNA polymerase II.. J Cell Sci.

[53] Iborra FJ, Pombo A, Jackson DA, Cook PR (1996). Active RNA polymerases are localized within discrete transcription ‘factories’ in human nuclei.. J Cell Sci.

[54] Cook PR (2002). Predicting three-dimensional genome structure from transcriptional activity.. Nature Genet.

[55] Klymenko T, Muller J (2004). The histone methyltransferases Trithorax and Ash1 prevent transcriptional silencing by Polycomb group proteins.. EMBO Rep.

[56] Pickersgill H, Kalverda B, de Wit E, Talhout W, Fornerod M, van Steensel B (2006). Characterization of the Drosophila melanogaster genome at the nuclear lamina.. Nat Genet.

[57] Krajewski WA, Becker PB (1998). Reconstitution of hyperacetylated, DNase I-sensitive chromatin characterized by high conformational flexibility of nucleosomal DNA.. Proc Natl Acad Sci USA America.

[58] Mellitzer G, Bonne S, Luco RF, Van De CM, Lenne-Samuel N (2006). IA1 is NGN3-dependent and essential for differentiation of the endocrine pancreas.. EMBO J.

[59] Sarma K, Nishioka K, Reinberg D (2004). Tips in analyzing antibodies directed against specific histone tail modifications.. Methods Enzym..

[60] Maestro MA, Boj SF, Luco RF, Pierreux CE, Cabedo J (2003). Hnf6 and Tcf2 (MODY5) are linked in a gene network operating in a precursor cell domain of the embryonic pancreas.. Human Mol Genet.

