# Supplementary figures and images for: Targeted Deficiency of the Transcriptional Activator Hnf1α Alters Subnuclear Positioning of Its Genomic Targets
Source: PLoS Genet. 2008 May 23;4(5):e1000079. doi: 10.1371/journal.pgen.1000079 (PMC2375116; doi:10.1371/journal.pgen.1000079)

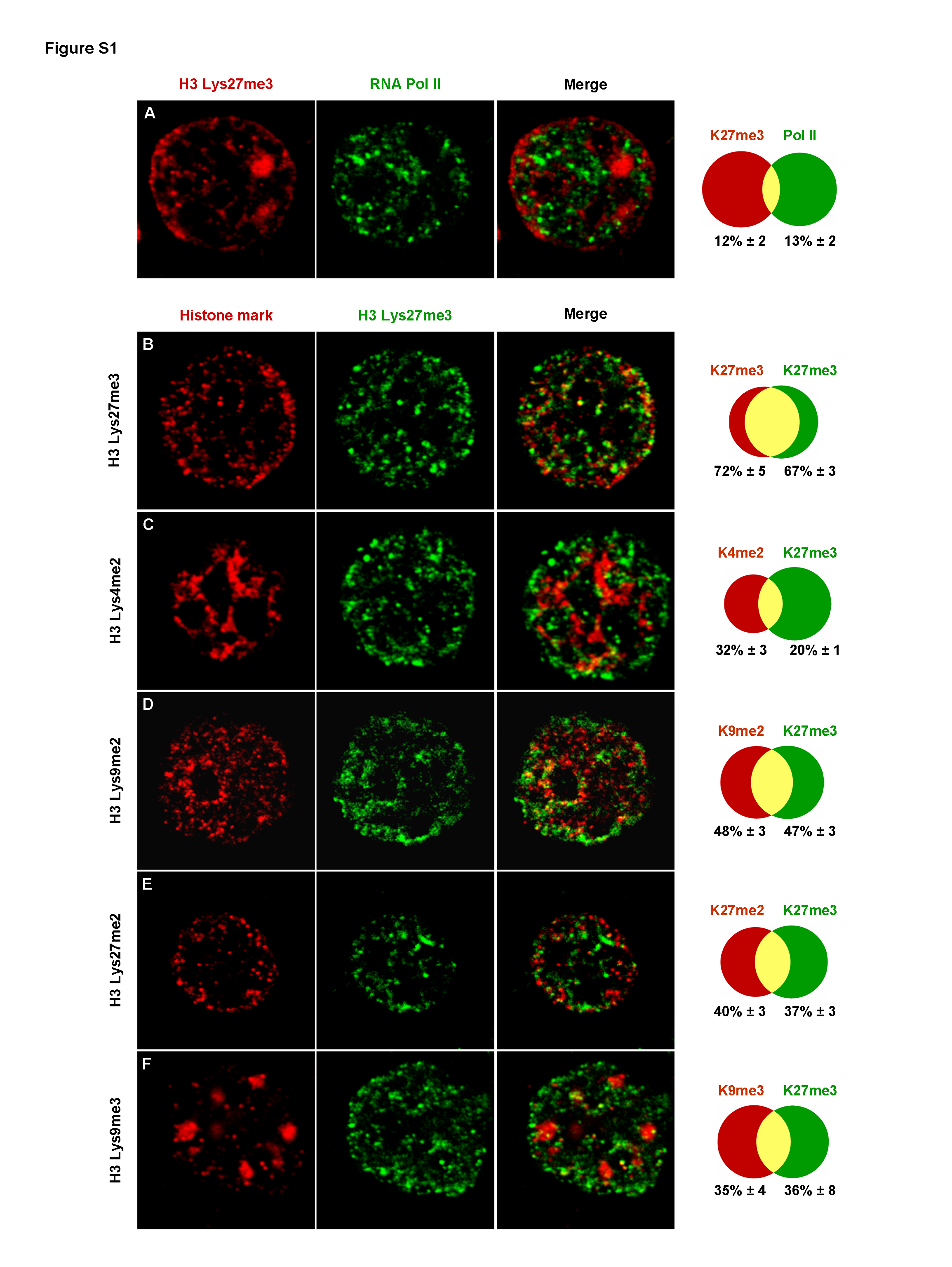

Supplement: Figure S1 — Immunofluorescence analysis of H3-Lys27me3 in hepatocytes. (A) Dual immunofluorescence analysis of RNA polymerase II (green) and H3-Lys27me3 (red) in hepatocytes after the immuno-FISH procedure showing that this process does not alter staining patterns. (B–F) Dual immunofluorescence analysis of hepatocytes with anti-H3-Lys27me3 (green) and either an alternate anti-Lys27me3 antibody (B), or anti-H3-Lys4me2 (C), anti-Lys9me2 (D), anti-Lys27me2 (E) or anti-Lys9me3 (F) antibodies (red). Colocalization analyses are depicted as Venn diagrams on the right side of immunolocalization images and were performed as described in the legend of Figure 2. (7.62 MB TIF) [file pgen.1000079.s001.tif]

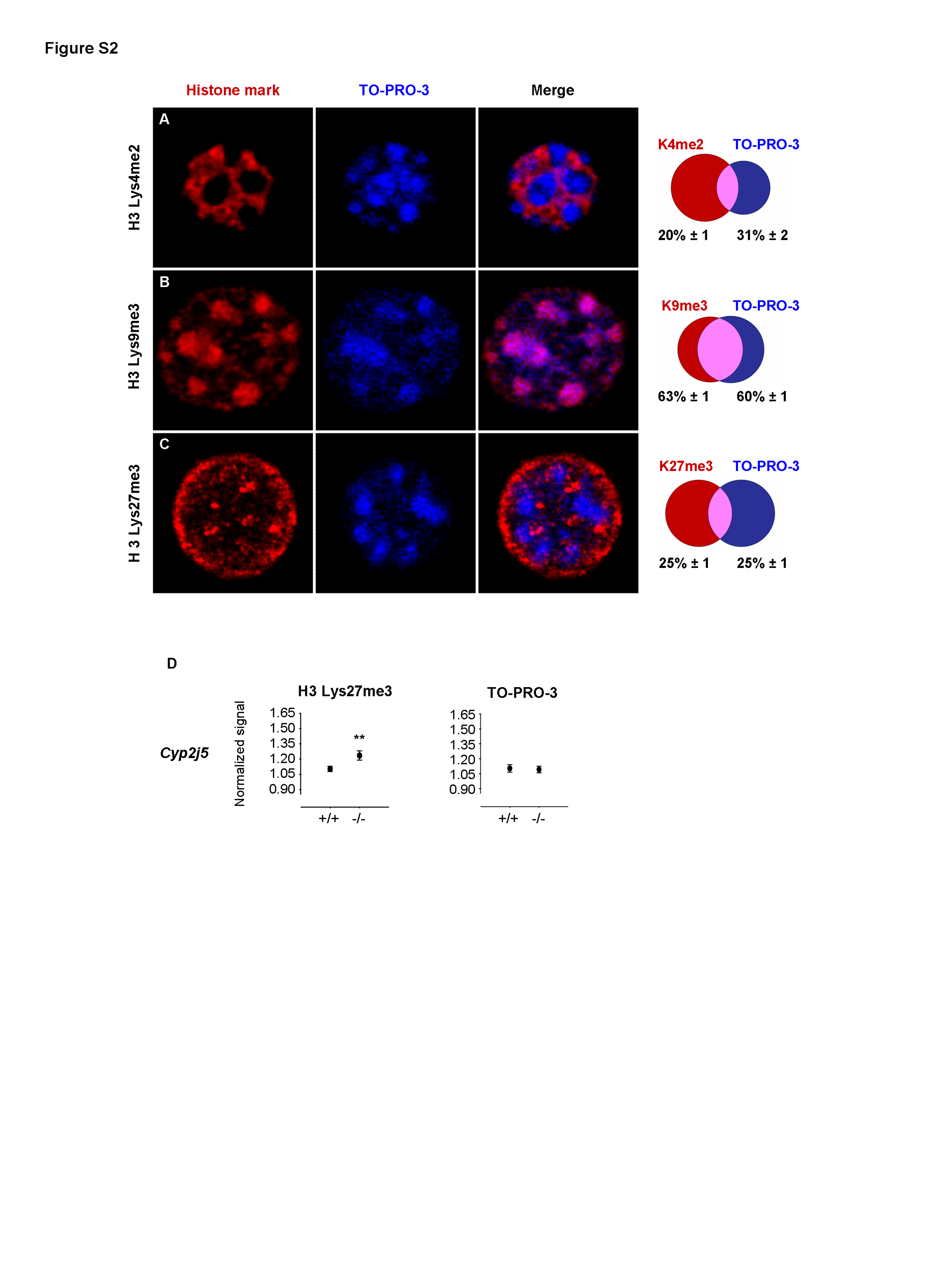

Supplement: Figure S2 — Analysis of the correlation between TO-PRO-3 density and histone modification patterns and gene positioning. (A–C) Immunofluorescence analysis of H3-Lys4me2 (A), H3 Lys9me3 (B), and H3-Lys27me3 (C) enrichment (red) compared with the DNA marker TO-PRO-3 (blue). Colocalization was analyzed and depicted with Venn diagrams as described in Figure 2. (D). Quantitative analysis of H3-Lys27me3 and TO-PRO-3 enrichment in Cyp2j5 loci in Hnf1a +/+ (+/+) and Hnf1a−/− (−/−) hepatocytes. Non-thresholded signal intensities of methylated histone marks or TO-PRO-3 were measured at 70-200 FISH alleles. To correct for cell to cell variability each value was divided by its nuclear median value, and is referred to as the normalized signal in the graphs. The graphs depict mean±SEM values. **P<0.01 relative to Hnf1a +/+ cells. (4.07 MB TIF) [file pgen.1000079.s002.tif]

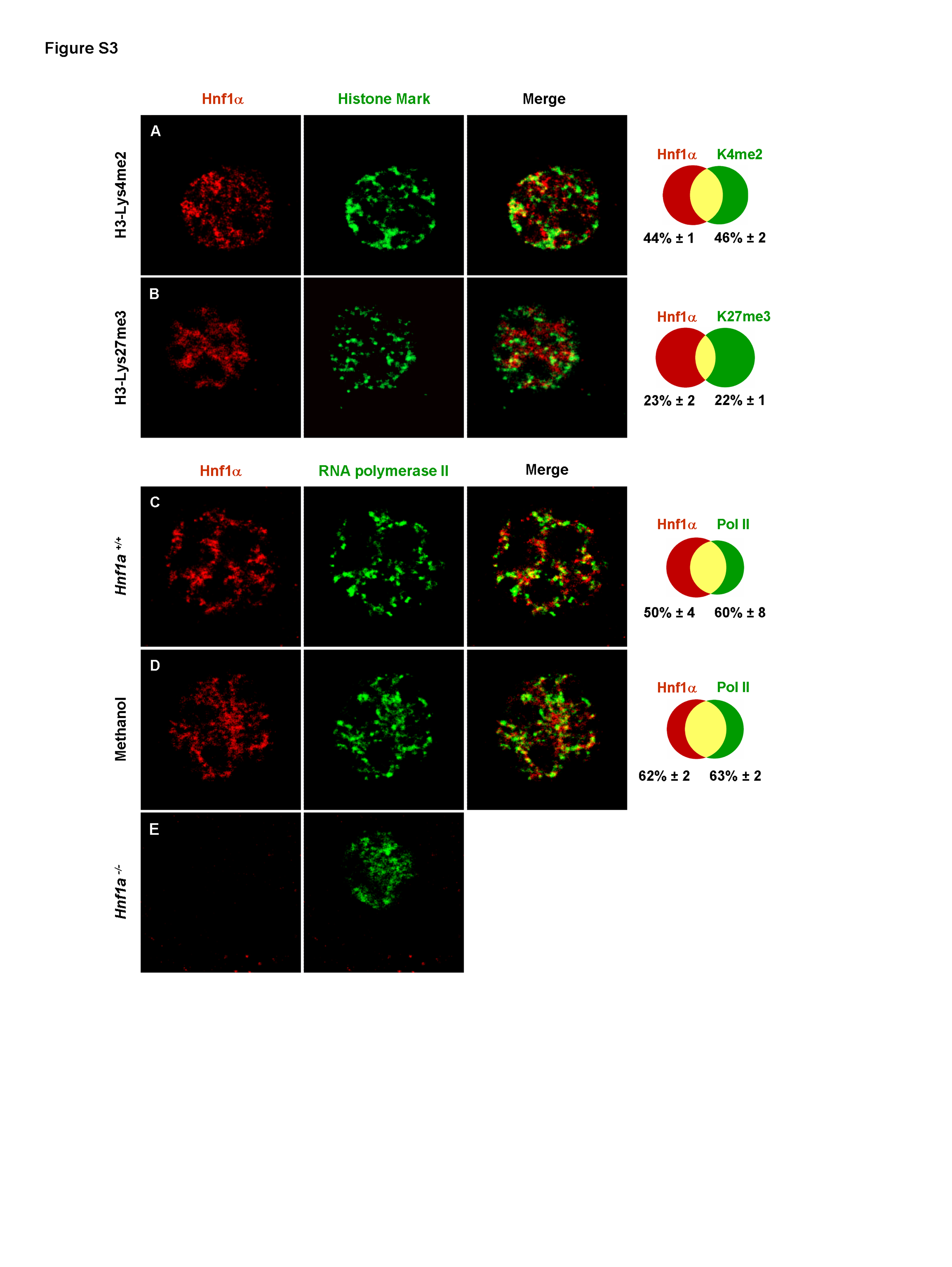

Supplement: Figure S3 — Subnuclear distribution of Hnf1α in histone code and RNA polymerase II domains. (A,B) Dual confocal immunofluorescence analysis of Hnf1α (red) and H3-Lys4me2 (A) or H3-Lys27me3 (B) (green) in interphase hepatocyte nuclei. (C–E) Dual immunofluorescence analysis of Hnf1α (red) and RNA polymerase II (green) in control hepatocytes fixed with 4% paraformaldehyde (C) or methanol (D) and in Hnf1a−/− hepatocytes fixed with 4% paraformaldehyde (E). Colocalization analysis was performed and Venn diagrams were arranged as described in the legend of Figure 2. (4.12 MB TIF) [file pgen.1000079.s003.tif]

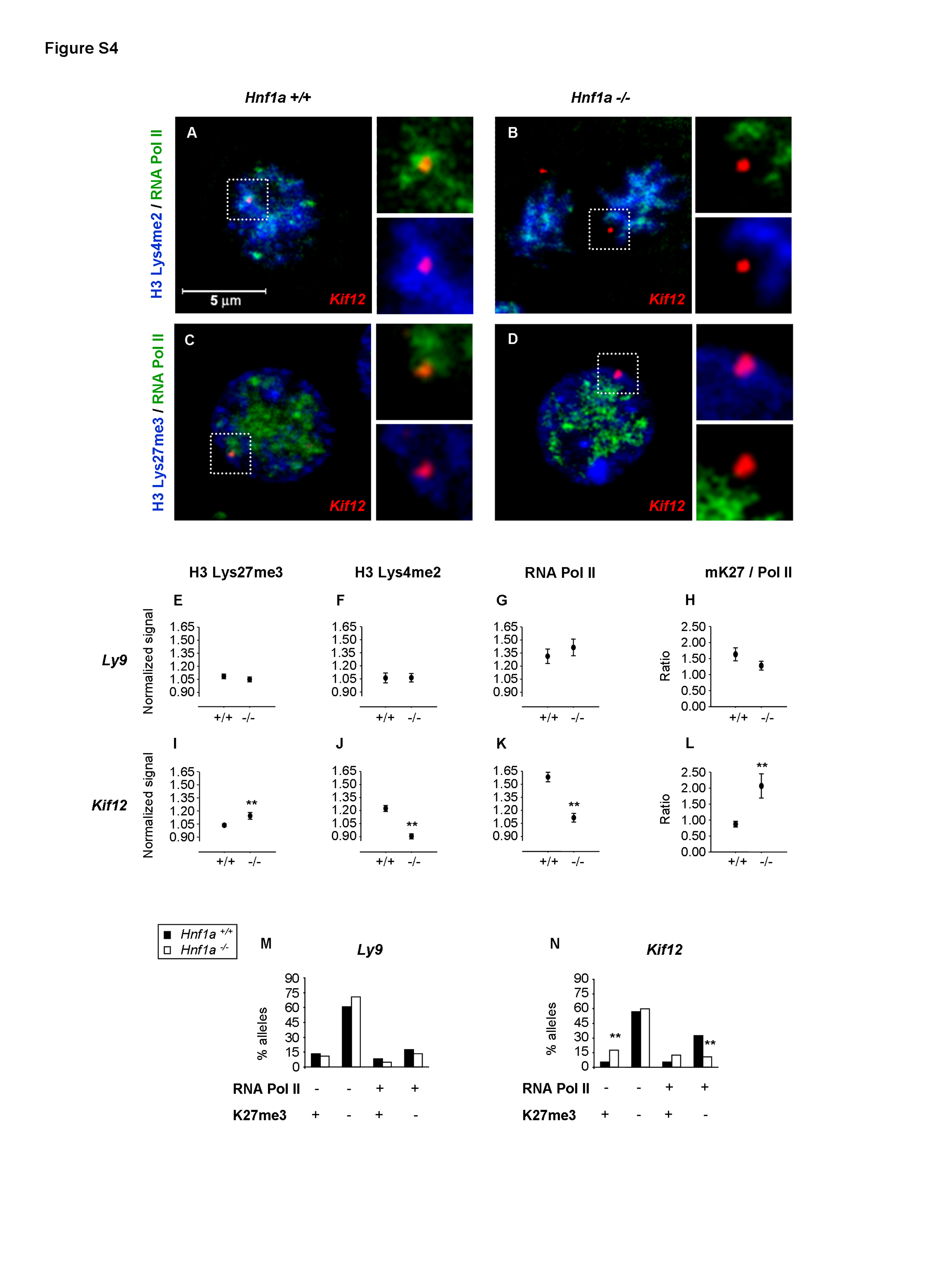

Supplement: Figure S4 — Hnf1α-dependent Kif12 activity in islet-cells correlates with differential positioning in RNA polymerase II and histone code domains. (A–D) Representative confocal immuno-FISH analysis in Hnf1a +/+ and Hnf1a−/− islet-cells of the Kif12 locus (red) with RNA polymerase II (RNA Pol II, green) and either H3-Lys4me2 (A,B) or H3-Lys27me3 (C,D) (blue). The framed regions containing FISH signals of Kif12 are shown at higher magnification on the right of each panel with omission of only blue or green channels. (E–L) Quantitative analysis of histone marks and RNA polymerase II in Kif12 (I–L) and control (Ly9, E–H) loci in Hnf1a +/+ and Hnf1a−/− islets. For each condition, non-thresholded signal intensities were measured at 100–200 FISH signals and each value was divided by the nuclear median intensity in the same channel. The graphs thus depict the average of such normalized signal values±SEM, except in H,L which shows mean±SEM of H3-Lys27me3/RNA polymerase II ratios (mK27/Pol II). (M,N) Classification of alleles from Ly9 control (M) and Kif12 (N) in 4 categories according to the simultaneous enrichment (+) or non-enrichment (−) of RNA polymerase II (RNA Pol II) and H3-Lys27me3 (K27me3) in Hnf1a +/+ (black bars) and Hnf1a −/− (white bars) islets as described in Figure 3O-P. Results are expressed as % of alleles for each genotype. **p<0.01 relative to Hnf1a +/+ cells using Mann-Whitney or Fisher's exact test as appropriate. (3.89 MB TIF) [file pgen.1000079.s004.tif]

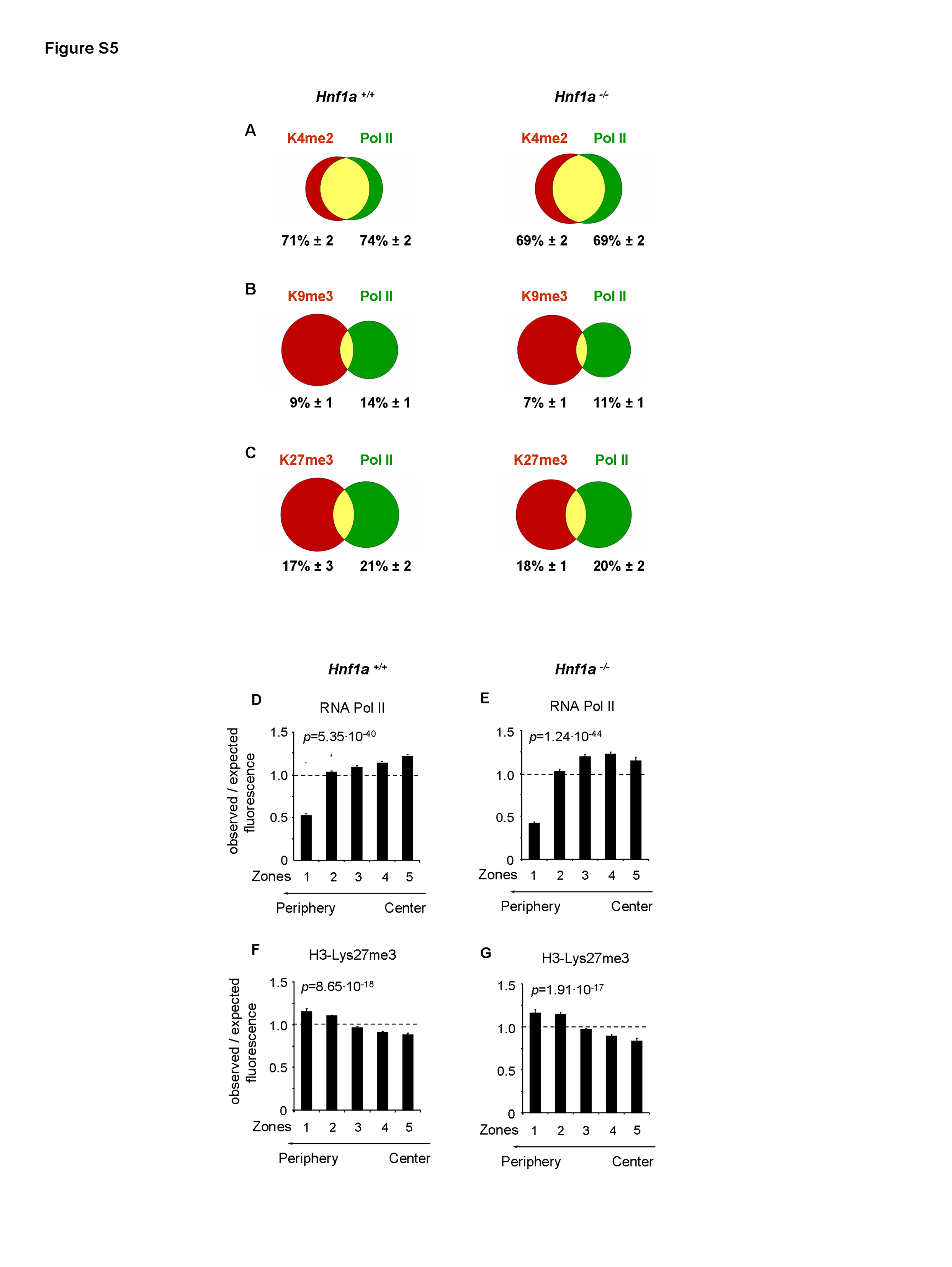

Supplement: Figure S5 — Histone modification/RNA Polymerase II colocalization and radial distribution patterns are similar in Hnf1a+/+ and Hnf1a−/− hepatocytes. (A–C) Venn diagrams showing the colocalization of H3-Lys4me2 (A), H3-Lys9me3 (B) and H3-Lys27me3 (C) with RNA polymerase II in interphase Hnf1a+/+ and Hnf1a−/− hepatocytes. The mean±SEM percentage of colocalizing pixels of 20 nuclei is shown below. (D–G). Erosion analysis of the nuclear distribution of RNA polymerase II (D, E), and H3-Lys27me3 (F,G) in interphase Hnf1a+/+ and Hnf1a−/− hepatocytes. Erosion analyses were performed as described in the legend of Figure 2. (0.61 MB TIF) [file pgen.1000079.s005.tif]

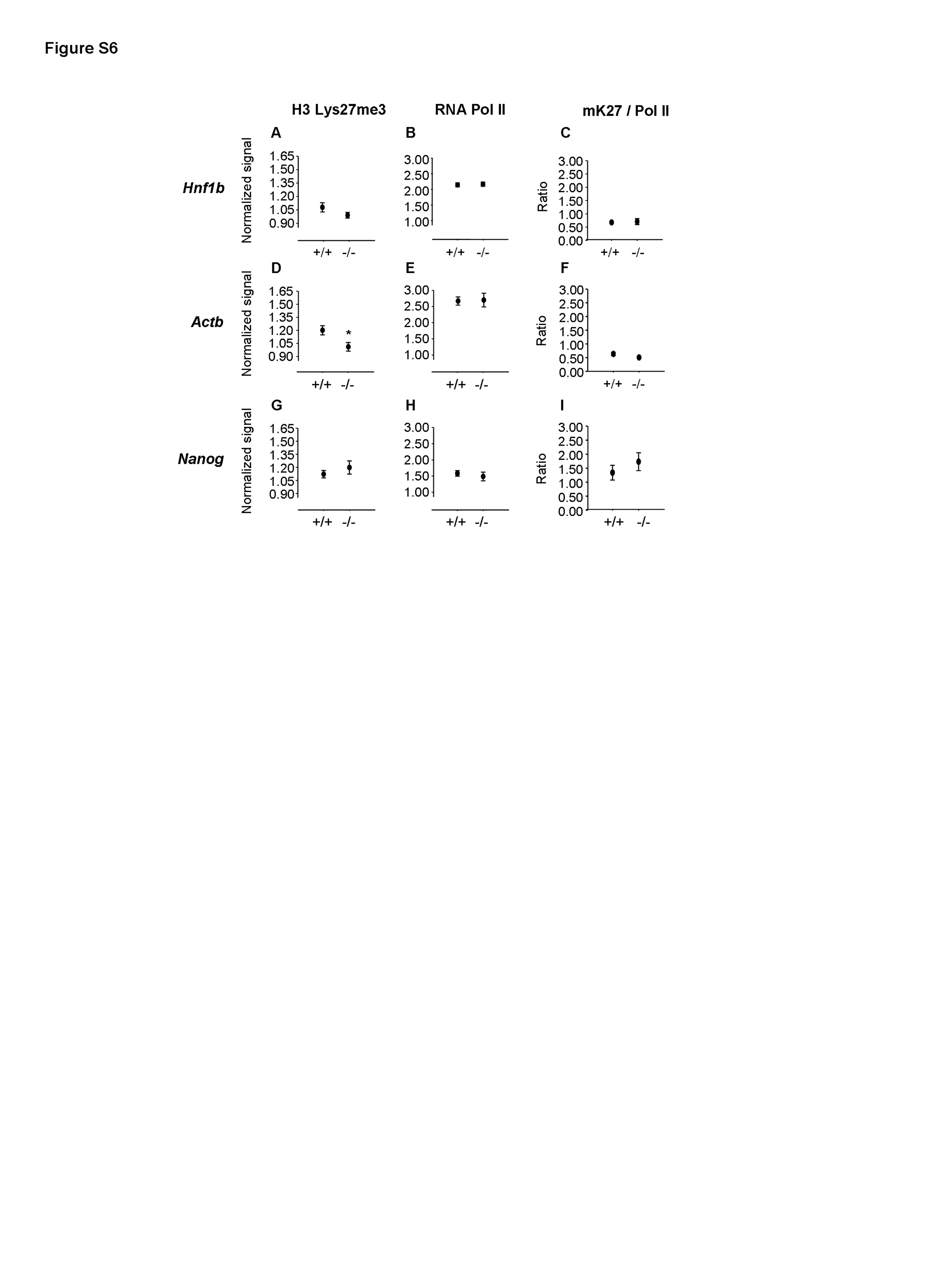

Supplement: Figure S6 — Quantitative analysis of histone marks and RNA polymerase II at control genes in Hnf1a +/+ and Hnf1a−/− hepatocytes by DNA immuno-FISH. Non-thresholded signal intensities were measured at 100-200 FISH signals and corrected by the nuclear median value (normalized signal) exactly as described in the legend of Figure 3. The graphs depict mean±SEM values. *P<0.05 relative to Hnf1a +/+ cells. (0.35 MB TIF) [file pgen.1000079.s006.tif]

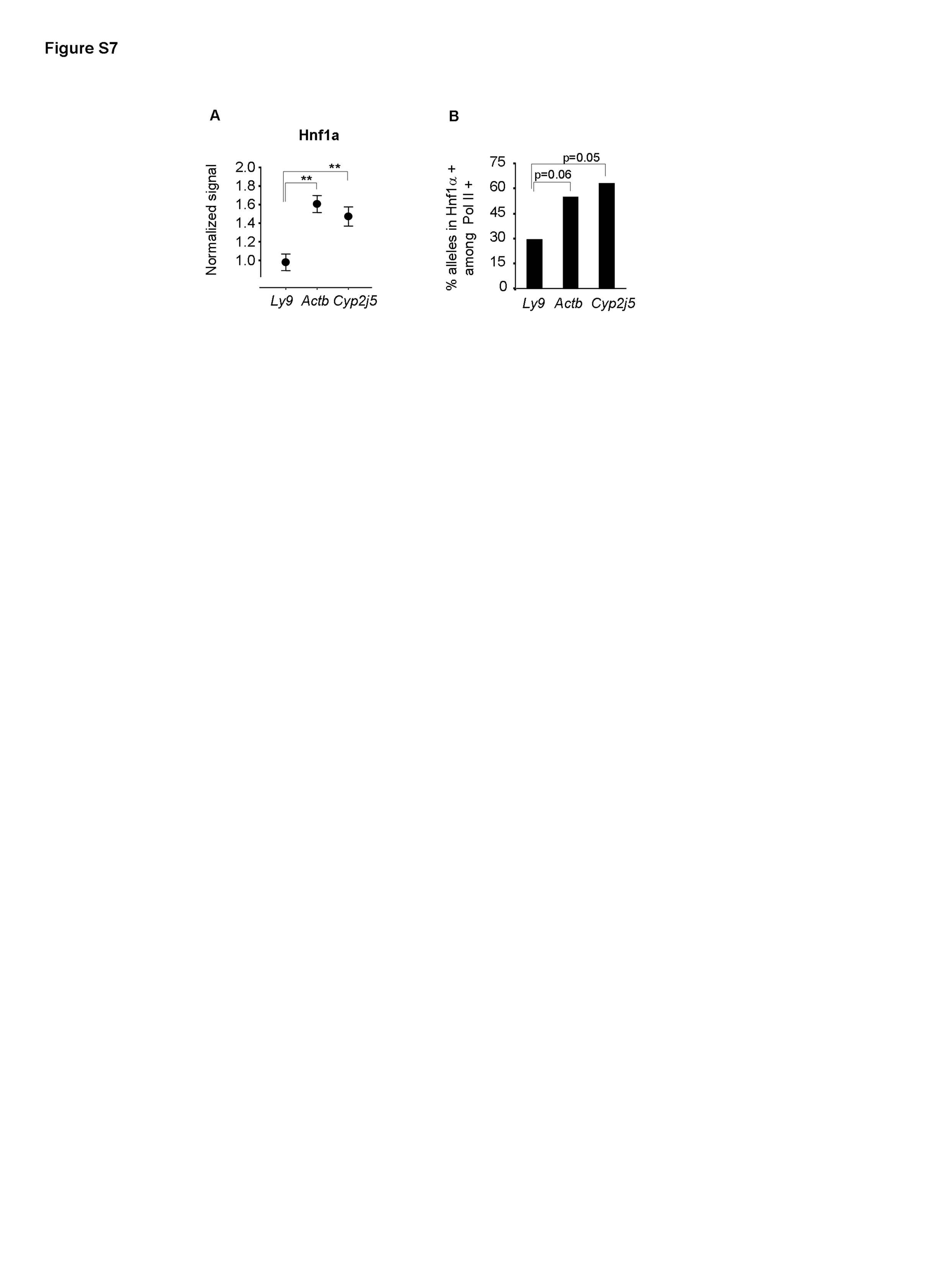

Supplement: Figure S7 — Active Hnf1α-dependent and independent loci preferentially localize in Hnf1α-rich domains. (A) Quantitative analysis of Hnf1α immunofluorescence signal intensity at the Hnf1α-target locus Cyp2j5, at a non Hnf1α-target active control gene (Actb), and at an inactive locus (Ly9) in wild-type hepatocytes. Immunofluorescence signals were normalized as described in Figure 3. (B) Percentage of alleles located in RNA polymerase II-rich domains that are also located in Hnf1α enriched domains in wild-type hepatocytes. Immunofluorescence signals were normalized and categorized essentially as described in Figure 3. *P<0.05 and **P<0.01 relative to Ly9. (0.22 MB TIF) [file pgen.1000079.s007.tif]

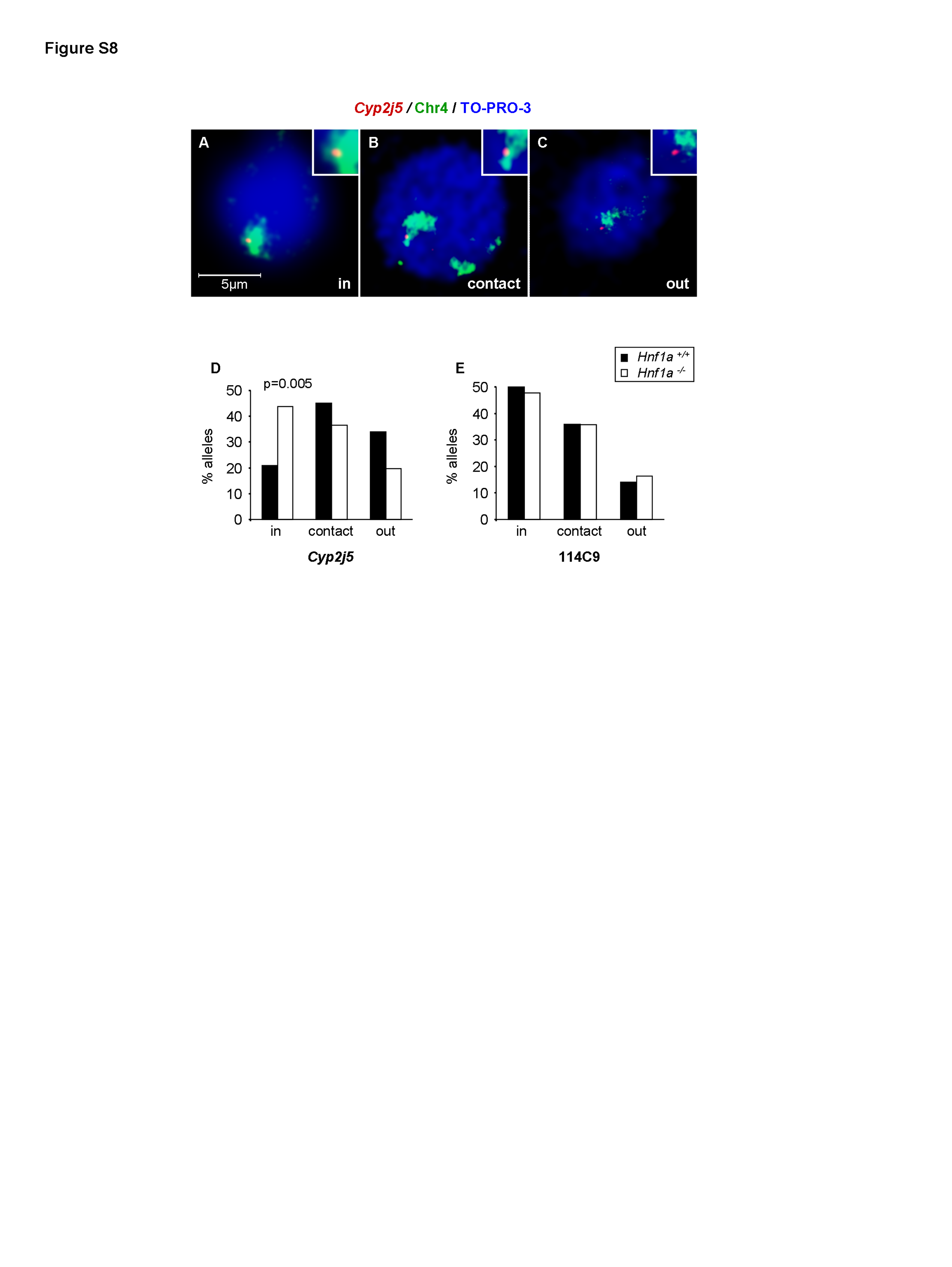

Supplement: Figure S8 — Hnf1α-deficiency causes altered positioning of Cyp2j5 relative to its chromosome territory. (A–C) Representative confocal images of Cyp2j5 DNA FISH (red) and chromosome 4 paint (green) in hepatocytes counterstained with TO-PRO-3 (blue) showing alleles that are classified as being either in, out or in contact of the chromosome territory. More details on the criteria for classification are described in methods. Higher magnifications of Cyp2j5 FISH signals are shown in the upper right panels. (D–E) Percentage of Cyp2j5 (D) and 114C9 (E) alleles that are located in, out, or in contact with the chromosome 4 territory in Hnf1a +/+ (black bars) and Hnf1a −/− (white bars) hepatocytes. Significance values for the comparison of allele distributions between Hnf1a +/+ and Hnf1a− /− hepatocytes were obtained by Fisher's exact test. (1.76 MB TIF) [file pgen.1000079.s008.tif]

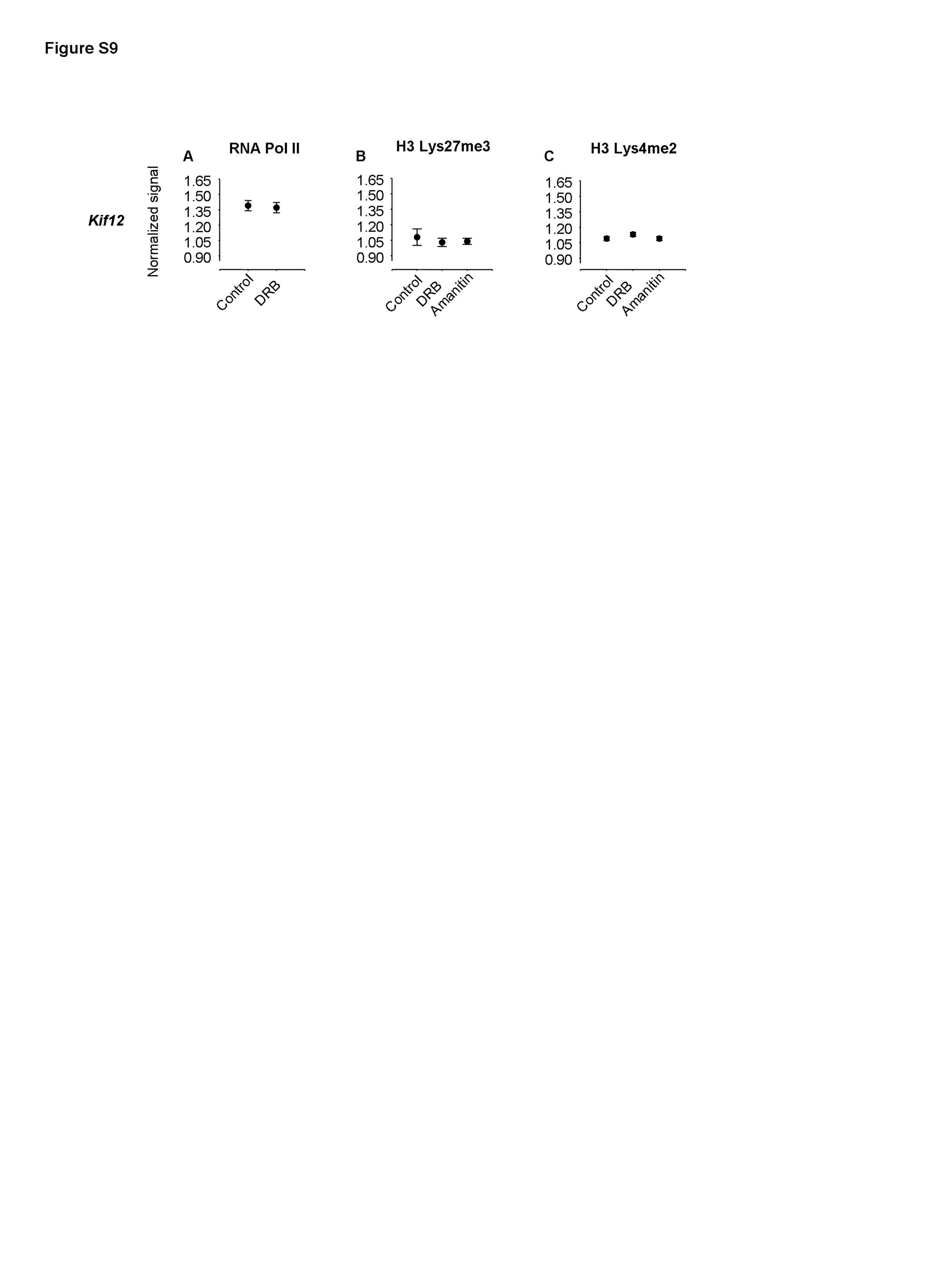

Supplement: Figure S9 — Inhibition of transcriptional activity does not modify Kif12 positioning in RNA polymerase II and histone code domains. Graphs show immuno-DNA FISH quantitation of H3-Lys4me2, H3-Lys27me3, and RNA polymerase II fluorescence at the Kif12 locus in MIN6 beta-cells treated for 4 hr with RNA polymerase II inhibitors α-amanitin (50 µg/mL) or 5,6-dichloro-1-beta-D-ribobenzimidazole (DRB) (20 µg/mL) vs. non-treated cells. Non-thresholded fluorescence signal intensities of histone marks and RNA polymerase II at 160-200 FISH signals were divided by the nuclear median intensity in the same channel, and are referred to as normalized signal in the graphs, essentially as described in Figure 3. The graphs depict mean±SEM values. Note that in the α-amanitin-treated cells RNA polymerase II fluorescence is not measured because this treatment results in marked reduction of RNA polymerase II foci. (0.24 MB TIF) [file pgen.1000079.s009.tif]
