# Supplementary material for: Targeted Deficiency of the Transcriptional Activator Hnf1α Alters Subnuclear Positioning of Its Genomic Targets
Source: PLoS Genet. 2008 May 23;4(5):e1000079. doi: 10.1371/journal.pgen.1000079 (PMC2375116; doi:10.1371/journal.pgen.1000079)
Supplement: Table S1 — Oligonucleotide sequences used in chromatin immunoprecipitation analysis. (0.03 MB DOC) [file pgen.1000079.s010.doc]

# Table S1. Oligonucleotide sequences used in chromatin immunoprecipitation analysis.

| **Gene** | **Forward** | **Reverse** | **Prod. Length**  **(bp)** | **Annealing (ºC)** |
| --- | --- | --- | --- | --- |
| Tbp | atcagatgtgcgtcaggcgtt | Tgcggagaaaatgacgcga | 102 | 60 |
| Actb (0bp) | gccccgcgtgtccctcaaac | Gctccgcgtcgctcactcacc | 383 | 60 |
| Actb coding seq | ggtgtggggagcaggttgg | Gtgggtgaagggctagatgtatgg | 649 | 55 |
| Nanog | agaggatgccccctaagcttt | Acagttaatcccacctgcagg | 103 | 60 |
| Ly9 | gcctgaatccaagaagaggaca | Ccagatcctgcaaggaaattgt | 140 | 55 |
| Afm | ctacctgtcaggcaagcacttt | Cggtcctggtgcaaatttcta | 255 | 55 |
| Pah | cattgccaggcctgtctgagc | Gttgccctgacgtagcagtgga | 193 | 60 |
| Cyp2j5 (0 bp) | gggcaggaaggggtgag | Tgacggcttctgaggatgtt | 214 | 60 |
| Cyp2j5 -2Kb | ggctcttacactttcctacaatgg | gccacagaggtcacaaaactattc | 155 | 55 |
| Cyp2j5 -1Kb | tccctcaatgtgagccaatg | Ttcatgcctaggacctatgacct | 108 | 55 |
| Cyp2j5 +1Kb | tcgctttatgacaccggttaga | Tgttcgaaaccactcacagga | 122 | 55 |
| Cyp2j5 +4Kb | acatctaaggcccacgtaccca | Gcaccaccgaggtccaatatt | 213 | 55 |
| Cyp2j5 +7Kb | ggcttctttcacagtgttcctca | Ttatgctcagtcttcaactctcgg | 137 | 55 |
| Cyp2j5 +13Kb | tcaacctcacaccagtcagaatg | Gaatcccaccaaccatgga | 106 | 55 |
| Cyp2j5 coding seq | tcctctgttttccctcttactcac | Tacatgccaccacctctcctac | 1296 | 55 |
