# Supplementary material for: Targeted Deficiency of the Transcriptional Activator Hnf1α Alters Subnuclear Positioning of Its Genomic Targets
Source: PLoS Genet. 2008 May 23;4(5):e1000079. doi: 10.1371/journal.pgen.1000079 (PMC2375116; doi:10.1371/journal.pgen.1000079)
Supplement: Table S2 — Bacterial artificial chromosomes used in this study. * Gene expression is represented as −, +, or ++ based on qualitative judgment of expression (not expressed, relative low expression, or relative high expression, respectively). ND: not determined. For genes marked in bold blue this information has been obtained experimentally by reverse transcription PCR or microarray expression analysis, while for genes marked in black this information has been collected from the Unigene EST Expression profile viewer (www.ncbi.nlm.nih.gov/entrez). #Cyp2j6 locus is immediately adjacent but not included in the BAC clone 263F12. Note that Hnf1β mRNA is mildly increased in Hnf1a−/− hepatocytes. (0.07 MB DOC) [file pgen.1000079.s011.doc]

| BAC name | Chr. Band | Size (Kb) | Gene symbol | Gene name | *Hnf1a^+/+^* hepatocytes* | *Hnf1a^-/-^* hepatocytes* | *Hnf1a^+/+^* pancreas* | *Hnf1a^-/-^* pancreas* |
| --- | --- | --- | --- | --- | --- | --- | --- | --- |
| RP23-117I23 | 6qF1 | 221.8 | *Dppa3* | developmental pluripotency-associated 3 | **-** | **-** | **-** | **-** |
|  |  |  | ***Nanog*** | **Nanog** | **-** | **-** | **-** | **-** |
|  |  |  | *Slc2a3* | solute carrier family 2 | + | + | **-** | **-** |
|  |  |  | *Foxj2* | forkhead box J2 | **-** | **-** | ++ | ++ |
| RP23-284L5 | 5qG2 | 194.1 | *Fbxl18* | F-box and leucine-rich repeat protein 18 | **-** | **-** | **-** | **-** |
|  |  |  | ***Actb*** | **actin, beta** | **++** | **++** | **++** | **++** |
|  |  |  | *Fscn1* | fascin homolog 1, actin bundling protein | + | + | ++ | ++ |
|  |  |  | *Ubce7ip1* | ubiquitin conjugating enzyme 7 interacting | **-** | **-** | + | + |
| RP23-7C5 | 11qC | 235.2 | *BC065092* | Riken, hypothetical ARM repeat fold containing | + | ND | ++ | ND |
|  |  |  | ***Hnf1b(Tcf2)*** | **Hepatocyte nuclear factor 1β** | **+** | **++** | **+** | **+** |
|  |  |  | *Ddx52* | DEAD (Asp-Glu-Ala-Asp) box polypeptide 52 | **-** | **-** | + | + |
| RP23-384B3 | 1qH3 | 204.7 | *Refbp2* | RNA and export factor binding protein 2 | **-** | **-** | + | + |
|  |  |  | *Itlna* | intelectin a | **-** | **-** | + | + |
|  |  |  | *Cd244* | CD244 natural killer cell receptor 2B4 | **-** | **-** | **-** | **-** |
|  |  |  | ***Ly9*** | **lymphocyte antigen 9** | **-** | **-** | **-** | **-** |
|  |  |  | *Slamf7* | SLAM family member 7 | **-** | **-** | + | + |
| RP23-12L1 | 4qC5 | 152.1 | *Cyp2j11* | cytochrome P450, family 2, subfamily j 11 | **-** | **-** | **-** | **-** |
| RP23-263F12 | 4qC5 | 125.4 | ***Cyp2j6^#^*** | **cytochrome P450, family 2, subfamily j 6** | **+** | **-** | **-** | **-** |
| RP24-277J5 | 4qC5 | 130.1 | ***Cyp2j9*** | **cytochrome P450, family 2, subfamily j 9** | **+** | **-** | **-** | **-** |
|  |  |  | ***Cyp2j5*** | **cytochrome P450, family 2, subfamily j 2** | **++** | **-** | **+** | **+** |
| RP24-68H9 | 4qC5 | 185.6 | ***68H9*** | **No genes or spliced ESTs** |  |  |  |  |
| RP24-114C9 | 4qC5-C6 | 200.9 | ***114C9*** | **No genes or spliced ESTs** |  |  |  |  |
| RP23-448J19 | 4qB3 - C1 | 192.1 | ***Ambp*** | **alpha 1 microglobulin/bikunin** | **++** | **-** | **++** | **-** |
|  |  |  | ***Kif12*** | **kinesin family member 12** | **-** | **-** | **++** | **-** |
|  |  |  | *Col27a1* | collagen type XXVII alpha 1 | **-** | **-** | ++ | + |
| RP24-288B11 | 5qE1 | 161.7 | ***Alb*** | **Albumin** | **++** | **+** | + | + |
|  |  |  | ***Afm*** | **Afamin** | **++** | **-** | - | - |
|  |  |  | *Afp* | alpha-feto-protein | ++ | ++ | + | + |
|  |  |  | *RassF6* | Ras association domain family 6 | + | + | + | + |

**Table S2**. Bacterial artificial chromosomes used in this study * Gene expression is represented as –, +, or ++ based on qualitative judgment of expression (not expressed, relative low expression, or relative high expression, respectively). ND: not determined. For genes marked in bold blue this information has been obtained experimentally by reverse transcription PCR or microarray expression analysis, while for genes marked in black this information has been collected from the Unigene EST Expression profile viewer (www.ncbi.nlm.nih.gov/entrez). ^#^*Cyp2j6* locus is immediately adjacent but not included in the BAC clone 263F12. Note that *Hnf1β* mRNA is mildly increased in *Hnf1a^-/-^* hepatocytes.
